# Optimal design of graphene-based plasmonic enhanced photodetector using PSO

**DOI:** 10.1038/s41598-024-65311-x

**Published:** 2024-07-03

**Authors:** Asghar Molaei-Yeznabad, Kambiz Abedi

**Affiliations:** Faculty of Electrical Engineering, Shahid Beheshti University, Tehran, 1983969411 Iran

**Keywords:** CMOS-compatible, Graphene, Photodetector, Plasmonic, Particle swarm optimization, Nanoscience and technology, Optics and photonics

## Abstract

In this paper, we report a graphene-based plasmonic photodetector optimized using the particle swarm optimization (PSO) algorithm and compatible with complementary metal–oxide–semiconductor (CMOS) technology. The proposed photodetector structure is designed to minimize fabrication challenges and reduce production costs compared to more complex alternatives. Graphene has been used for its unique properties in the detection region, titanium nitride (TiN) as a CMOS-compatible metal, and both to aid in plasmonic excitation. Photodetectors have key parameters influenced by multiple independent variables. However, practical constraints prevent thorough adjustment of all variables to achieve optimal parameter values, often resulting in analysis based on several simplified models. Here we optimize these variables by presenting a new approach in the field of photodetectors using the capabilities of the PSO algorithm. As a result, for the proposed device at the wavelength of 1550 nm, the voltage responsivity is 210.6215 V/W, the current responsivity is 3.7213 A/W, the ultra-compressed length is less than 3$\mu {\text{m}}$, and the specific detectivity is 2.566×${10}^{7}$ Jones were obtained. Furthermore, the device in question works under the photothermoelectric effect (PTE) at zero bias and has zero dark current, which ultimately resulted in a very low noise equivalent power (NEP) of 4.5361 ${\text{pW}}/\sqrt{\text{Hz}}$.

## Introduction

### Basic review

Today, the possibility of commercializing systems and devices in a completely optical way, due to the existing limitations, especially the integration of optical devices, is facing serious and numerous challenges. Therefore, providing conditions that can effectively solve these challenges is strongly felt. Silicon-based optical devices and their integration with silicon-based electronic devices can be introduced as a suitable solution that can cause transformation in different fields and have many applications in different industries, such as Refs.^1–3^:

- *High-Speed Data Communication:* In industries such as telecommunications, data centers, and high-performance computing, the integration of optoelectronic devices with silicon enables high-speed data transmission.
- *Optical Sensing and Imaging:* Integrating optoelectronic devices with silicon enables advanced sensing and imaging capabilities in applications such as medical diagnostics, environmental monitoring, and industrial inspection. Optoelectronic sensors can detect light, temperature, pressure, and other environmental parameters with high sensitivity and accuracy.
- *Photonic Integrated Circuits (PICs):* Integration of optoelectronic devices with silicon facilitates the development of PICs, which combine optical and electronic components on a single chip. Integrating optoelectronics with silicon-based electronics presents several challenges, primarily due to the differing nature of these technologies and examining these factors can be useful^4–6^.
- *Material Incompatibility:* Silicon, the predominant material in traditional electronics, is not inherently suitable for light emission or detection. Optoelectronic devices typically use materials like gallium arsenide (GaAs), indium phosphide (InP), or organic semiconductors, which have different properties compared to silicon.
- Fabrication Techniques: The manufacturing processes for silicon-based electronics and optoelectronic devices are often incompatible. Silicon chips are typically fabricated using techniques like photolithography, which may not be directly applicable to optoelectronic materials. Integrating different fabrication methods on the same chip can be complex and costly.
- Thermal Management: Optoelectronic devices can generate significant heat during operation. Integrating them with silicon-based electronics requires careful thermal management to prevent overheating, which can degrade performance and reduce device lifespan.
- Scalability and Cost: Scaling up production of integrated optoelectronic devices while maintaining cost-effectiveness is a challenge. The specialized materials and fabrication processes used in optoelectronics can be more expensive than those for silicon-based electronics, potentially limiting widespread adoption.
- Compatibility with Existing Infrastructure: Integrating optoelectronics with silicon-based electronics also requires compatibility with existing infrastructure, such as communication networks and data centers. Ensuring interoperability and seamless integration with established systems is crucial for practical applications.
- Size difference: The photonic diffraction limit imposes challenges for integrating optical components with CMOS technology, as the size scales of photonic components and CMOS transistors differ significantly.

Solutions such as using materials compatible with silicon, introducing simple structures in order to reduce manufacturing costs, and using the plasmonic effect to overcome the size difference between the photonic diffraction limit and CMOS technology are among the solutions that respond to some of these problems^4,7–9^, which is examined in the following cases. Most of the photodetectors used in silicon photonics are germanium-based^10–12^, but this type of photodetector has limitations in integrating with CMOS technology^13,14^. Currently, various materials are used in the manufacture of photodetectors, including silicon-based photodetectors^15–22^, gallium arsenide-based photodetectors^23–26^, aluminum gallium nitride-based photodetectors^11,17,27^, indium gallium arsenide-based photodetectors^28,29^, indium arsenide-based photodetectors^30^, and photodetectors based on a combination of different materials^31^ are among these cases. However silicon-based photodetectors are of double importance due to their compatibility with CMOS technology^32^. Using metals compatible with CMOS technology, such as TiN, also seems to be a very good option for excitation of surface plasmon polariton (SPPs)^4,11,12,20,33^. Surface plasmon polaritons are electromagnetic excitations that exist at the interface between a metal and a dielectric (or semiconductor). The plasmon that travels along the interface between the dielectric and the metal in the form of a moving wave is called a SPP^18,34^. These unique electromagnetic excitations lead to a significant increase in the electromagnetic field at the metal–dielectric interface, so that light can be confined beyond its diffraction limit, which is a great advantage. One of the promising approaches is to engineer the plasmonic environment around two-dimensional materials to regulate the light-matter interaction^35^. Two-dimensional materials have emerged as a captivating area of research due to their unique properties and promising applications across a multitude of fields. Since the discovery of the first two-dimensional material, various types of these amazing materials have been introduced. Among the widely used two-dimensional materials that have been used in various fields, the following can be mentioned^36–40^:

- *Transition Metal Dichalcogenides (TMDs):* Such as ${\text{MoS}}_{2}$ and ${\text{WSe}}_{2}$, which have strong intralayer bonding and weak interlayer interactions due to their layered structure and offer promising applications in optoelectronics.
- *Phosphorene:* A single layer of black phosphorus, which has attracted considerable attention due to its unique structure and tunable band gap. Due to its anisotropic electrical and optical properties, phosphorene can be suitable for applications such as field-effect transistors.
- *Hexagonal Boron Nitride (hBN):* This material has a hexagonal network structure similar to graphene but consists of alternating boron and nitrogen atoms. hBN exhibits excellent thermal and chemical stability, high electrical insulation, and low friction properties, making it an ideal substrate for graphene-based devices and a promising material for nanoelectronics and photonics.
- *MXenes:* They are a group of two-dimensional materials that have received attention in recent years due to their unique properties and promising applications. Due to their high surface area, MXenes can provide abundant active sites for electrochemical reactions, making them ideal candidates for electrodes in energy storage devices.
- *Graphene:* The fifth and most important case is the two-dimensional material graphene, whose discovery in 2004 sparked a renaissance in the study of two-dimensional materials and prompted researchers to further investigate the properties of this amazing material. Graphene has a remarkable atomic structure consisting of a single layer of carbon atoms densely arranged in a hexagonal lattice. Each carbon atom in graphene forms covalent bonds with three neighboring atoms, resulting in a very stable and strong structure. This arrangement provides extraordinary properties such as exceptional mechanical strength, high thermal conductivity, and remarkable electrical conductivity. In addition to the mentioned cases, these materials have far better advantages than other two-dimensional materials, among which the following can be mentioned:
- High carrier transfer rate^7,26,35,41^
- Wide responsivity range^27,37^
- Small footprint^16,19^
- Simple fabrication process^35^
- Low power consumption^19^
- Ultra-sensitivity^19,42^
- Compatibility with CMOS^13^

Also, graphene's unique optical and electronic properties make it highly suitable for photodetection applications. Firstly, due to its zero bandgap, graphene exhibits a broad spectral response, spanning from ultraviolet to infrared wavelengths^43^. This allows graphene-based photodetectors to operate across a wide range of frequencies, enabling versatility in applications. Secondly, graphene's high carrier mobility and low noise characteristics contribute to its excellent sensitivity and fast response time in detecting photons^44^. Electrons in graphene can move freely, facilitating efficient photoconversion and signal detection. Additionally, graphene's high carrier mobility enables rapid charge transport, leading to quick device response times. Furthermore, graphene's transparency and flexibility are advantageous for constructing photodetection devices with enhanced performance and versatility. Moreover, we can functionalize graphene's surface to enhance its light absorption properties or tune its electronic structure, thereby enhancing its performance in photodetection applications^20^. Additionally, by combining graphene with other materials to form heterostructures, we can design tailored photodetection devices with specific properties. Graphene is highly compatible with silicon due to its atomic structure and similar lattice constant to silicon. This compatibility allows graphene to be integrated seamlessly with silicon-based devices and manufacturing processes. Graphene can be grown directly on silicon substrates through techniques like chemical vapor deposition (CVD) or transferred onto silicon wafers, enabling straightforward integration into existing semiconductor fabrication processes^45^. Despite its compatibility, challenges remain in optimizing the interface between graphene and silicon to minimize defects, enhance electronic performance, and ensure long-term reliability. Controlling the doping and strain in graphene layers on silicon substrates is crucial for tailoring electronic properties and achieving desired device performance. However, all these factors make graphene an attractive material for photodetection applications. A photodetector designed using the unique properties of graphene can have better performance parameters than other types of photodetectors^38^. It should be noted that when this amazing material is used in two or more layers in combination with other materials, it shows extraordinary properties, which again makes it an attractive material for use in photodetectors. Photodetectors designed and characterized using bilayer graphene have shown extremely high performance, which makes them suitable in the infrared regime and applications that have high sensitivity^46^. The photodetectors designed using this material have high responsivity, high detectivity, and extremely low NEP, which distinguishes them from other similar photodetectors^47,48^.

In many applications and the operational mode, it is not possible to improve all the parameters, in this case, a trade-off must be created between the parameters. With this work, the parameters that are of great importance can be optimized and an ideal photodetector can be found that is suitable for that application. To trade off the available parameters, traditional or non-traditional methods can be used to find the best values. A traditional method usually starts with an initial solution and converges to the optimal solution with each successive iteration. This convergence depends on the choice of initial approximation. Having complete information about the objective function, long execution time, and low adaptability in facing continuous problems are among the limitations of these methods. Therefore, the need for non-traditional methods is felt. Metaheuristic algorithms are high-level problem-solving strategies that provide approximate solutions to optimization problems. Unlike traditional exact optimization methods that guarantee finding the optimal solution, metaheuristic algorithms focus on efficiently exploring the solution space to find good-quality solutions within a reasonable amount of time, especially for complex or large-scale optimization problems where exact methods may be impractical. Genetic algorithms, ant colony optimization, and PSO are examples of powerful meta-heuristic algorithms that are used in many applications due to their capabilities. Inspired by the processes of natural selection and evolution, genetic algorithms employ mechanisms such as selection, crossover, and mutation to iteratively evolve a population of candidate solutions toward better solutions. The power of genetic algorithms lies in their ability to efficiently explore large solution spaces, especially when the problem landscape is complex, nonlinear, or high-dimensional. The foraging behavior of real ants, where they collectively find the shortest path between their colony and a food source by depositing and following pheromone trails, inspires ACO. Artificial ants in ACO solve optimization problems by traversing paths in the solution space. Based on information from pheromone trails, ACO effectively explores the solution space by balancing exploration (searching for new solutions) and exploitation (exploiting known solutions)^50^. But, one of the most important, most widely used and most powerful existing non-traditional methods is the PSO algorithm. PSO algorithm is a Swarm-based meta-heuristic stochastic optimization technique proposed by Eberhart and Kennedy in 1995^51,52^. This non-traditional optimization algorithm does not have the limitations of traditional algorithms, and this has caused this algorithm to cover a wide range of problems and be used^53^. The PSO algorithm offers several advantages that make it a popular choice for solving optimization problems, especially engineering problems^53–55^:

- *Simplicity:* PSO is relatively easy to implement and understand compared to some other optimization algorithms. Its simplicity makes it accessible to a wide range of users, including those without extensive optimization expertise.
- *Efficiency:* PSO is computationally efficient, especially for optimization problems with continuous or real-valued variables. It requires minimal parameter tuning and can converge to good solutions quickly, making it suitable for problems with time constraints.
- *Robustness:* PSO is robust to variations in problem formulations and solution spaces. It can handle non-linear, multimodal, and noisy optimization problems effectively without requiring explicit knowledge of the problem's gradients or constraints.
- *Parallelism:* PSO inherently supports parallel computation, allowing for efficient implementation on parallel and distributed computing architectures. This parallelism accelerates the optimization process, particularly for large-scale optimization problems.
- *Global Search Capability:* PSO has the ability to explore the entire solution space efficiently, enabling it to find globally optimal or near-optimal solutions. The swarm's exploration and exploitation capabilities facilitate the discovery of diverse regions of the solution space, leading to robust and high-quality solutions.
- *Adaptability:* PSO is adaptable to various problem domains and can be easily customized to suit specific optimization tasks. By adjusting parameters such as swarm size, inertia weight, and acceleration coefficients, PSO can be tailored to different problem characteristics and objectives.
- *Versatility:* PSO has been successfully applied to a wide range of optimization problems across diverse domains, including engineering. Its versatility and effectiveness make it a valuable tool for researchers and practitioners in various fields.

The PSO algorithm offers several advantages compared to genetic algorithms (GAs) and ant colony optimization (ACO): Simplicity and Ease of Implementation Fewer Parameters to Tune, Efficient Exploration of Solution Space, Computational Efficiency, Robustness to Parameter Settings and Parallelism are among these things. In this work, we theoretically investigate a graphene-based plasmonic photodetector optimized using the PSO algorithm and present the results. In the present work, four key parameters are considered for the proposed photodetector to check the performance of the device. These parameters include voltage and current response, photodetector length, special detection, and noise-equivalent power. As will be explained in the methodology section, each of these parameters depends on a number of independent variables according to their descriptive equations. Each of these variables also has a numerical range due to the continuity of their values. This is while each parameter mentioned may depend on two, three, or more independent variables at the same time. A large number of these variables are considered constant in the tasks that are usually done in order to reduce the computational and practical complications. But this is despite the fact that if there is a method that can simultaneously search all the numbers in the defined ranges for the variables and finally choose the most optimal numbers, then it can be said that the value calculated for the specified parameter has its most optimal state using these variable values. This work is done in this article by the PSO algorithm. In fact, the main goal is to optimize the noise equivalent power parameter of the proposed photodetector, which is done by the PSO algorithm, and it is called single-objective optimization. But on the other hand, the point that needs to be noticed is that the other mentioned parameters are also dependent on these variables and when these variables are optimized, they will have positive effects on these parameters and will improve them as well, which in this article has also been done and the term quasi-multi-objective has been used. However, the review of different types of works that have been done in the field of graphene-based plasmonic photodetectors can provide a better view of the importance of this type of photodetector. On the other hand, considering that the PSO meta-heuristic algorithm is used in this article to optimize the photodetector parameters, a review of similar works can be effective in highlighting the importance of the work done. Therefore, in the following, a comprehensive review of recent works has been carried out.

### Literature review

Al-Aloul et al.^4^ introduced a groundbreaking graphene-based photodetector. By integrating graphene with CMOS microelectronics and silicon photonics, they achieved high responsivity using CMOS-compatible TiN. This plasmon-enhanced photodetector has a responsivity of $\text{0.7717 }{\text{A}}/{\text{W}}$, flat response across the telecom C-band, and ultra-compact dimensions of just 3.5 µm. Remarkably, it operated at zero-bias, consumed no energy, and showed an extremely low noise equivalent power of approximately $\text{20 }{\text{pW}}/\sqrt{\text{Hz}}$. Yu et al.^22^ unveiled p-n homojunction graphene photodetectors (GPDs), renowned for zero-bias operation and wide bandwidth. Departing from tradition, they demonstrated high-performance silicon-integrated GPDs capable of efficiently absorbing light at 1.55 and 2 μm wavelengths. Their analysis revealed remarkable responsivity, exceeding $\text{2.78 }{\text{V}}/{\text{W}}$, and bandwidths surpassing 22 GHz, all under zero bias. With a linear dynamic range spanning over 24 dB, these GPDs offered a promising frontier for silicon photonics, extending beyond the confines of 1.55 μm for applications in optical communications and photonics. Vangelidis et al.^32^ explored the realm of PICs, seeking efficient and swift light detection compatible with Si processing. They championed graphene-based photodetectors for their promise in meeting these demands, thanks to their unique properties. Despite challenges, they unveiled an unbiased asymmetric configuration, harnessing plasmonic enhancement to achieve $\sim\text{0.}{6} \, {\text{A}}/{\text{W}}$ responsivity, $\text{22 }{\text{pW}}/\sqrt{\text{Hz}}$ noise equivalent power, and 100 GHz operation speed at zero power consumption. Through rigorous modeling and experimentation, they paved the way for efficient, fast, and versatile photodetection within PICs. Viti et al.^38^ achieved room temperature terahertz detection using large-area single-layer graphene (SLG) grown by CVD. They integrated SLG into antenna-coupled field effect transistors, exploring different substrate terminations (${\text{SiO}}_{2}$, ${\text{Al}}_{2}{{\text{O}}}_{3}$) and encapsulation layers (${\text{HfO}}_{2}$ or large-area CVD-grown hexagonal boron nitride). Their scalable architectures yielded response times of ~ 5 ns and noise equivalent powers of $\text{1 }{\text{nW}}/\sqrt{\text{Hz}}$ under zero-bias operation, highlighting SLG's thermoelectric properties for photodetection. Xie et al.^39^ introduced a groundbreaking approach to PTE detectors by utilizing doped polyaniline (PANI)/graphene composites on poly(ethylene terephthalate) substrates. They simplified fabrication through tip sonification and magnetic stirring, achieving a highly sensitive, bias-free photodetector with a peak detectivity of $\text{6.8}\times {10}^{7} {\text{Jones}}$ and a responsivity of $\text{2.5 }{\text{V}}/{\text{W}}$. The composite demonstrated remarkable flexibility, enduring over 300 bending cycles, and exhibited rapid, high-performance photovoltage responses, mimicking human interactions. This innovation promised broad applications in nondestructive health monitors, photodetectors, and wearable IoT devices. Schuler et al.^56^ introduced a groundbreaking advancement in graphene integrated photonics, offering superior performance compared to conventional Si photonics. They overcame the challenge of low responsivity in GPDs by integrating a photo-thermoelectric GPD with a Si microring resonator. Achieving > 90% light absorption in a ~ 6 μm SLG channel along a Si waveguide under critical coupling, they generated a voltage responsivity of ~ $\text{90 }{\text{V}}/{\text{W}}$,. This innovation enabled GPDs to operate at a ${10}^{-{9}}$ bit-error rate, rivaling mature semiconductor technology, without the need for transimpedance amplification. This breakthrough promised a reduction in energy-per-bit, cost, and footprint, marking a significant leap in photonics technology. Li et al.^57^ pioneered a breakthrough in thermal photodetection by blending methylamine lead iodide perovskite (${\text{MAPbI}}_{3}$) with graphene oxide to form a composite material. This blend, boasting a high temperature coefficient of resistance (TCR) of $-\text{ 0.89\% }{\text{K}}^{-1}$ and low heat capacity at room temperature, enabled the creation of a sensitive and rapid IR to THz bolometer. The resulting ${\text{MAPbI}}_{3}$/graphene oxide detector achieved remarkable performance, with a responsivity of $\text{805 }{\text{mA}}/{\text{W}}$ and a response time of about $\text{700 }\mu {\text{s}}$ at 220 GHz, surpassing pure graphene oxide detectors by an order of magnitude. This innovative composite engineering opened new frontiers in THz detector technology. Yan et al.^58^ innovatively combined silicon photonics and graphene to create a double slot structure, addressing the low responsivity in current hybrid graphene/silicon photodetectors. By optimizing structural parameters, they enhanced graphene absorption while minimizing metallic absorption within the waveguide. This structure achieved a remarkable responsivity of $\text{603.92 }{\text{mA}}/{\text{W}}$ and a broad bandwidth of 78 GHz, offering a competitive solution for silicon photodetection. Additionally, their approach held promise for enhancing light-matter interaction in a wide range of hybrid two-dimensional material/silicon devices. Liu et al.^59^ developed a plasmon-enhanced few-layer ${\text{MoS}}_{2}$ photodetector on a gallium nitride substrate using a bowtie equal grid antenna structure. Through chemical vapor deposition, they achieved large-scale growth of few-layer ${\text{MoS}}_{2}$, significantly enhancing performance. The photodetector exhibited a high responsivity of $\text{0.82 }{\text{A}}/{\text{W}}$, low NEP of $\text{6.58}\times {10}^{-14} \, {\text{W}}/\sqrt{\text{Hz}}$, and detectivity of $\text{1.56}\times {10}^{12} {\text{Jones}}$ under 365 nm at 5 V bias, with a rise/fall time of 18/10 ms. This plasmonic structure offered a promising method to enhance photoresponse in other high-efficiency photoelectric devices. Ma et al.^60^ showcased a breakthrough in photonic technology with their plasmonic slot graphene photodetector on a silicon-on-insulator platform. They achieved a high responsivity of $\text{0.7 }{\text{A}}/{\text{W}}$ with just a 5 μm device length, surpassing conventional limitations. Through dual-lithography, they carved out 15 nm narrow slots, resulting in a 30-fold increase in responsivity per unit device length compared to traditional photonic graphene photodetectors. Additionally, their investigation revealed the dominance of channel-doping contributions over back-gated electrostatics, leading to quasi charge neutrality and unprecedented nonambipolar transport characteristics. These compact and efficient photodetectors paved the way for short-carrier pathways in next-generation photonic components and provided insights into short-channel carrier physics in graphene optoelectronic devices. Bansal et al.^61^ presented three photodetectors with different structures. The modeling performed led to the characterization of precise optoelectronic properties. The dual photodetectors covered a wide spectral response from UV to near-infrared wavelengths, and the heterogeneous dual broadband photodetector showed fast switching of the photocurrent. At a bias of $-\text{ 0.5 V}$ bias and 300 K, the highest external quantum efficiency, photocurrent responsivity, specific detectivity, and the lowest NEP of 71%, $\text{0.28 }{\text{A}}/{\text{W}}$, $\text{4.2}\times {10}^{12} {\text{Jones}}$, and $\text{2.59}\times {10}^{-17}\text{ W}$ was obtained at the wavelength of 480 nm, respectively. On the other hand, given that this article uses the PSO over-the-counter algorithm to improve the performance of the proposed photodetector, a review of recent articles conducted in this field can give a better insight into this type of work that has recently received a lot of attention. Anjum et al.^62,63^ exploited machine learning techniques to optimize a modified uni-traveling-carrier photodetector for low phase noise at a 5 V bias. Comparing the metaheuristic algorithms such as particle swarm, genetic, and surrogate optimization algorithms, they found that PSO yielded the lowest phase noise, surpassing the current design by $\text{4.4 }{\text{dBc}}/{\text{Hz}}$. Their analysis revealed that the optimized design eliminated electrical bottlenecks, offering insights into enhanced photonic device performance through machine learning. Nag et al.^64^ demonstrated an optimization strategy for balanced photodetectors (BPDs) on a generic InP platform, crucial for high-speed coherent receivers at 1550 nm. Using PSO, they fine-tuned BPD design parameters to achieve maximum bandwidth while maintaining optimal responsivity. Their approach involved establishing an equivalent circuit model and addressing major bottlenecks like carrier transit time and RC loading. The algorithm yielded multiple design parameter combinations with identical output characteristics, offering flexibility. Additionally, they outlined a methodology for integrating lasers with optimized BPDs, facilitating the implementation of coherent receivers. Ferhati et al.^65^ proposed a novel MSM-UV-photodetector (PD) design based on dual wide band-gap material engineering, aimed at achieving high-performance self-powered operation. They developed analytical models to characterize the device properties, incorporating the impact of DM aspect on photoelectrical behavior, which were validated through numerical simulations. Their design modification effectively modulated the device's electric field, enabling efficient carrier separation and dark current reduction without an external voltage. Additionally, they introduced a hybrid approach combining analytical modeling and PSO to optimize device performance at zero bias. The optimized PD exhibited high responsivity $\text{98 }{\text{mA}}/{\text{W}}$ and superior ${\text{I}}_{\text{ON}}/{\text{I}}_{\text{OFF}}$ ratio (480 dB), making it suitable for low-cost self-powered applications in optical communication and monitoring. Djeffal et al.^66^ developed a cost-effective multispectral PD using an a-Si/Ti multilayer structure, achieving high UV–Visible-NIR photoresponse. They employed a novel design strategy combining Finite Difference Time Domain (FDTD) with GA to optimize the multilayer geometry. The fabricated PD exhibited 80% broadband absorbance across UV and NIR spectra (200–1100 nm) and demonstrated improved responsivity under UV, Visible, and NIR lights ($\text{1.9 }{\text{A}}/{\text{W}}$ at 365 nm, $\text{1.24 }{\text{A}}/{\text{W}}$ at 550 nm, and $0.\text{93 }{\text{A}}/{\text{W}}$ at 900 nm), with a high ${\text{I}}_{\text{ON}}/{\text{I}}_{\text{OFF}}$ ratio of 68 dB. This broad multispectral photodetection capability offered promising prospects for future cost-effective optoelectronic systems. Bencherif et al.^67^ suggested a way to make a 4H-SiC/thin film PD faster and better at sensing by combining the light trapping formalism with meta-heuristic optimization. They fixed the shadowing issue by using graphene electrodes and adding silver nanoparticles to improve the absorption of photons in the 4H-SiC active region. Their study investigated the effect of plasmonic engineering agents on PD properties. The proposed device achieved impressive performance metrics, including a maximum responsivity of $\text{269.6} \, {\text{m}}{\text{A}}/{\text{W}}$, a high photocurrent-to-dark current ratio (PDCR) of $\text{1.7}\times {10}^{6}$ and and a detectivity of $\text{5.4}\times {10}^{13} {\text{Jones}}$ Jones. In addition, their optimal design using the Multi-Objective Genetic Algorithm (MOGA) balances sensing performance and response time, achieving superior responsivity of $\text{564.5 }{\text{m}}{\text{A}}/{\text{W}}$ and detection $\text{1.25}\times {10}^{14} {\text{Jones}}$. This approach demonstrated significant improvements in the photodetector's sensitivity. Simsek et al.^68^ investigated solutions for using meta-heuristic algorithms in photodetector design. They showed that they could design high-performance photodetectors using modern optimization techniques. Subsequently, all designs generated during these optimization studies were then used to train a two-stage physics-inspired neural network to achieve better-performing devices.

### Novelty and contributions

This paper presents an innovative approach and concept in the realm of optimizing photodetectors by leveraging the capabilities of the PSO algorithm and integrating it with a graphene-based plasmonic photodetector. The photodetector presented in this study incorporates various physical sections, including a waveguide, metal electrodes, graphene, waveguide sidewalls, and a substrate. Additionally, this device encompasses several operational parameters, such as temperature, bias voltage, incident optical power, waveguide dimensions (length, width, and height), distance between electrodes, and photodetector dimensions (length, width, and height). Some parameters are considered to decrease the fixed complexity, while other conditions, such as waveguide dimensions, were optimized using alternative ways to facilitate the TM mode. One of the prominent features of the proposed photodetector is its simple geometry, which leads to an easy manufacturing process and thus reduces production costs compared to similar samples. On the other hand, by studying the characteristics of different materials used in photodetectors, two-dimensional graphene material was chosen as the main detection material. This material can increase the performance of the photodetector due to having a different process for stimulating its plasmons compared to the plasmons of metals and its high tunability by applying impurities. On the other hand, because this material has special intrinsic properties such as high electrical and thermal conductivity, zero effective mass, long emission length without scattering at room temperature, and high carrier mobility, it has been able to introduce itself as a suitable candidate for new generation photodetectors. The ability to integrate with CMOS technology is also one of the most important features of this photodetector, which is considered by using metals compatible with this technology such as TiN. The primary objective of this article is to investigate the theory and optimization of six crucial variables of the photodetector. This work is done by using the PSO algorithm with a quasi-multi-objective approach, with the aim of simultaneously reducing the noise equivalent power and the length of the photodetector while increasing the response and special detection. In this new approach that is proposed in this article, in the first step, the concept of particles in the PSO algorithm is considered equivalent to a certain value for the noise-equivalent power parameter of the photodetector. In the next step, the concept of global search in the PSO algorithm is considered equivalent to a global search in all defined intervals for six variables. In the final step, the concept of the cost function, which should be minimized in the PSO algorithm, is used, and it is considered equivalent to the noise equivalent power. This parameter is also desirable to be minimized as much as possible. Considering these definitions as well as practical limitations in theoretical studies, noise equivalent power is implemented as a cost function in the PSO algorithm. The PSO algorithm, taking into account all the restrictions and conditions determined for the six main variables, obtains the lowest value or, in other words, the most optimal possible value for NEP and determines a true value for each of these variables. By setting these values for the mentioned variables in operational mode, the most optimal value for NEP was achieved. It should be noted that the initial conditions defined for the variables have been chosen considering operational limitations so that more practical solutions can be obtained by the PSO algorithm. On the other hand, according to the relationship of noise equivalent power with the response parameters, the length of the photodetector and special detection, as well as the presence of the aforementioned variables in the relationships describing these parameters, it has caused optimal values for these parameters to be obtained. So, the novelty and contributions of this work will be outlined as follows:

- The use of a simple geometry for the proposed photodetector structure with the ability to easier fabrication and reduce production costs compared to similar complex samples.
- The use of two-dimensional graphene material due to its unique properties such as high electrical and thermal conductivity, adjustable plasmons using doping, zero effective mass, long diffusion length without scattering at room temperature, and high carrier mobility.
- Using metals compatible with CMOS technology such as TiN, in order to provide a photodetector that can be integrated with this technology.
- Introducing a new and conceptual approach by combining a powerful optimization algorithm such as PSO with an important optical device such as a photodetector.
- Carrying out complete theoretical investigations on how to implement the noise equivalent power objective function on the PSO algorithm.
- Precise and intelligent adjustment of the range of variables and their application in the PSO algorithm, taking into account the trade-off between them.
- Applying operational limits in the PSO algorithm to obtain the practical values of the parameters.
- A significant reduction in the amount of noise equivalent power and the length of the photodetector compared to the previous similar work.
- Significant increase in response and special detection of this photodetector compared to the previous work.
- Optimized design for operation at the 1550 nm telecommunication wavelength.
- Providing ultra-compressed detection length with optimal adjustment by the PSO algorithm.
- Performance under the influence of the PTE phenomenon and having zero bias.
- Improvement of response, special detection, and reduction of power equivalent noise compared to the previous similar work.

### Paper organization

This paper is organized as follows: After the introduction, Sect. 2 is devoted to describing the structure of the device. Section 3 is dedicated to the methodology used in this work and describes the governing equations of this tool and its explanations. In Sect. 4, additional explanations are mentioned under the title of methods, including the techniques used to design the waveguide of the photodetector, graphene absorption, carrier transfer mechanisms, and other values required in the article. Section 5 is devoted to results and discussions. In this section, the results obtained through optimization for the photodetector are mentioned in three separate subsections. In the first sub-section, the optimal values of the variables are calculated; in the second sub-section, the optimal values of the voltage response, current, and special detection are calculated and compared with the main reference; and the performance criteria of the proposed photodetector are compared with those of other photodetectors. Finally, the presented article will have a conclusion section.

## Device structure

**Figure 1:**
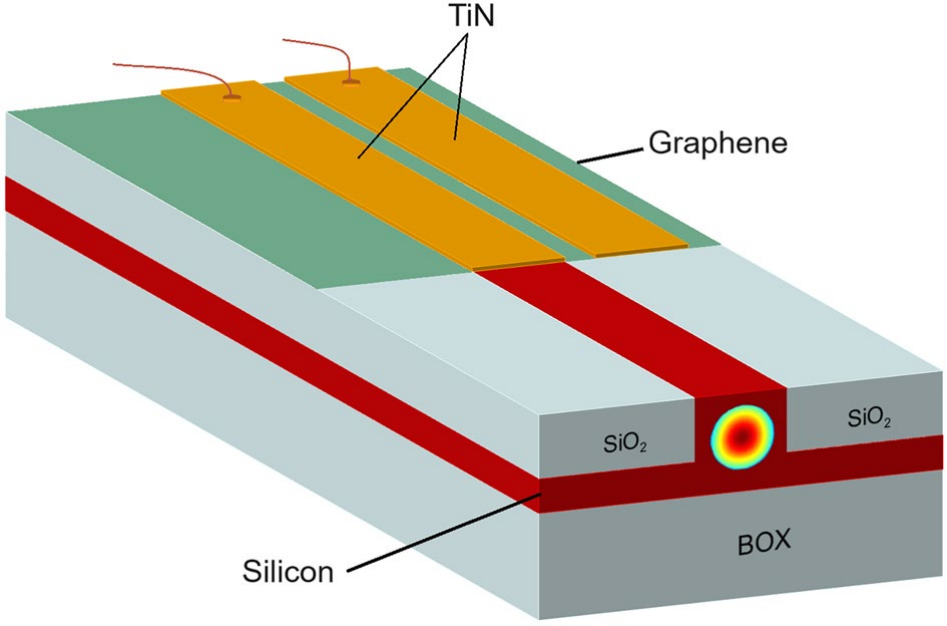
Overview of the photodetector device.

According to Fig. 1, the overview of the proposed device is shown. A silicon rib waveguide on top of the 2 µm in 0.5 µm bottom oxide layer (BOX) directs the incoming light to the detection part at the end of the optical link, where the optical signal is converted into its electrical format. In the detection part and on the sides of the waveguide, silicon dioxide has been used to facilitate the placement of graphene on the waveguide. Two TiN contacts placed on the graphene collect the electrical signal generated. In addition to signal collection by the central TiN film, this film Plasmonically enhances the interaction of the TM propagation mode with graphene due to the waveguide support of the TM mode, which increases the near field and as a result, increases the optical absorption of graphene and leads to a high optical response of the device. It should be noted that the dimensions related to the thickness of the silicon slab, the dimensions of the silicon waveguide, the thickness of the TiN contacts, the dimensions of the sides of the waveguide, refractive indices, and other constant values used in reference^69^ were also used in this work. Is. Therefore, it is recommended to see the reference mentioned to see the details and get more information. It should be noted that the dimensions related to the thickness of the silicon groove, the dimensions of the silicon waveguide, the thickness of the TiN contacts, the dimensions of the sides of the waveguide, the refraction coefficients and other constant values used in reference^69^ are mentioned and are also used in this article. Therefore, it is recommended to see the reference to see the details and get more information.

The originality of a structure, such as an photodetector or a method that has been used, is of vital importance in several ways and can be checked. Regarding the originality of a method, it has already been described, but in this section, the originality of the photodetector structure has been examined in the form of bullet points.

- *Enhanced Performance:* An original structure can lead to significant improvements in the photodetector’s performance. This includes higher sensitivity, faster response times, and greater accuracy in detecting light. Innovations in structure can optimize the interaction between light and the photodetector material, thereby enhancing its efficiency and effectiveness. In this regard, the proposed structure has been selected after reviewing valid articles and works that can eventually lead to construction and have shown proper performance.
- *Technological Advancement:* Original structures contribute to technological progress. They push the boundaries of current knowledge and capabilities, leading to the development of more advanced and sophisticated photodetectors. This, in turn, can catalyze further research and innovation in the field. The integration of several advanced technologies such as CMOS technology, plasmonic effect and two-dimensional graphene material together can unconsciously lead to the development of technology to meet the needs.
- *Integration with New Technologies:* Original designs can facilitate the integration of photodetectors with emerging technologies. For instance, advances in materials science, such as the use of graphene or other nanomaterials, can be more effectively utilized through innovative structural designs.

The proposed structure presents itself as a highly suitable option for related applications for several compelling reasons. Firstly, its simplicity ensures ease of understanding and implementation, which can significantly reduce the time for fabrication. Secondly, the availability of materials used in this structure means that the materials can be readily supplied. Thirdly, the technology required for the construction of this structure is already widely available, ensuring that no specialized or rare equipment is needed, which facilitates broader adoption and integration into existing systems. Finally, the reproducibility of this structure is a critical advantage and indeed represents the ability to repeatedly produce an electronic component or system with the same performance and characteristics in all versions. Together, these features make the proposed structure an attractive and practical choice for various applications within its field. In this work, an implementable structure is used along with an optimization algorithm. This is while providing a new insight into the topic of optimization of optical devices, which is the main goal of this work. There are many structures that can be built and put into operation. There are also many optimization algorithms that have extremely high power in solving various problems. Providing a complete and comprehensive approach in order to open a path for integrating the advantages of optimization algorithms and important structures of optoelectronics and reducing limitations is one of the main goals of this article.

## Methodology

Optimization is a fundamental aspect of scientific research and development, which aims to achieve the best possible results in various fields. Using optimization, one can easily solve complex problems, optimize resource allocation, improve processes, and discover optimal solutions that cannot be achieved through traditional approaches. Therefore, many optimization algorithms including continuous^49,51–53,70–72^ or discrete^73^ have been introduced for various applications. The PSO algorithm is one of the most famous and powerful algorithms for solving continuous problems due to its simplicity, adaptability, global search capability, and fewer assumptions, which can be used in many subjects, especially engineering. One of the most important engineering issues is the discussion of photodetectors. Due to their importance in a wide range of applications, these optical devices, such as imaging devices^74,75^, optical communication^17,26^, environmental monitoring^10,17^, and Medical diagnoses^76,77^ play an important role. Therefore, the optimization of these optical devices is very important. Photodetectors can be optimized in several ways. Material optimization, device structure optimization, performance conditions optimization, and noise optimization are four very important issues in the discussion of photodetectors that can be optimized using optimization algorithms. Optimization in operating conditions ensures that the photodetector can perform at its best in different applications and lighting conditions. In this article, optimization of device structure, optimization of operating conditions, and optimization of NEP have been used. The basic idea behind the PSO algorithm is to create a computational approach that can optimize the trajectory of multiple particles in a multidimensional space. In this work, we write the variables in the NEP relationship based on all the variables that depend on them. Each of these variables can be assumed as a dimension. With more variables, we will have a multi-dimensional space that this algorithm can easily search in this space and get the optimal value. One of the features of the PSO algorithm compared to other algorithms is that it is simple and has few descriptive equations^51–53^:

1 $${\nu }^{\text{i}}\left[\text{t+1}\right]= \text{ w} {\nu }^{\text{i}}\left[{\text{t}}\right] \, \text{ + }{\text{c}}_{1}{{\text{r}}}_{1}\left({\text{x}}^{\text{i,best}}\text{[t]-}{\text{x}}^{\text{i}}\text{[t]}\right)+ \text{ } {\text{c}}_{2}{{\text{r}}}_{2}\left({\text{x}}^{\text{g,best}}\text{[t]-}{\text{x}}^{\text{i}}\text{[t]}\right)$$

where w is the inertia coefficient,${\text{c}}_{1}$ is the personal learning coefficient,${\text{c}}_{2}$ is the collective learning coefficient, ${\text{r}}_{1}$ and ${\text{r}}_{2}$ are random numbers obtained from the normal distribution, ${\upnu }^{\text{i}}$ is the speed of the ${\text{i}}^{\text{th}}$ particle, ${\text{x}}^{\text{i}}$ is the personal best memory, and ${\text{x}}^{\text{g}}$ is the collective best memory.

2 $${\text{x}}^{\text{i}}\left[\text{t+1}\right]\text{=}{\text{x}}^{\text{i}}\text{[t]+}{\nu }^{\text{i}}\left[\text{t+1}\right]$$

It should be noted that the coefficient of inertia from the ${\text{w}}\in \text{ [0.4, 0.9]}$ interval and the personal and collective learning coefficients are also selected from the ${0}\le {\text{c}}_{1} \, \& \, {\text{c}}_{2}\le {2}$ interval according to the problem. In addition to the conventional coefficients whose values were mentioned, another type of these coefficients was introduced by Mr. Clerc called contraction coefficients, which had a series of important features, some of which are specified as bullet points. In addition to the conventional coefficients, whose values were mentioned, another type of coefficient was introduced by Clerc called constriction coefficients^78^. These coefficients in the PSO algorithm are used to control the speed of particles in the crowd and ensure the stability and convergence of the algorithm. These coefficients have a series of important features that make them suitable for use in the present work. In the following, some of the most important features of these coefficients and their describing equations are mentioned.

- *Velocity Control:* The constriction coefficient is applied to the velocity update formula to regulate the speed at which particles move through the search space. It prevents particles from accelerating too quickly, which can lead to instability and divergence.
- *Stability and Convergence:* The primary purpose of the constriction coefficient is to improve the stability of the PSO algorithm. By moderating the velocities, it ensures that particles do not diverge or oscillate excessively, promoting smoother convergence to the optimal solution.
- *Comparison to Inertia Weight:* The constriction coefficient is an alternative to the inertia weight approach, which also aims to control particle velocity. While the inertia weight method uses a single parameter to scale the velocity.
  $$\chi =\frac{2}{\phi \text{-2+}\sqrt{{\phi }^{2}\text{-4}\text{.} \, \phi }} {\phi }_{1}, {\phi }_{2}>{0} \& \, \phi {\triangleq \phi }_{1}+ {\phi }_{2}$$
  3 $$\text{w= }\chi , {\text{c}}_{1}= \text{ } \chi \text{ . } \, {\phi }_{1}\text{, }{\text{c}}_{2}= \text{ } \chi \text{ . }{\phi }_{2}$$

**Figure 2:**
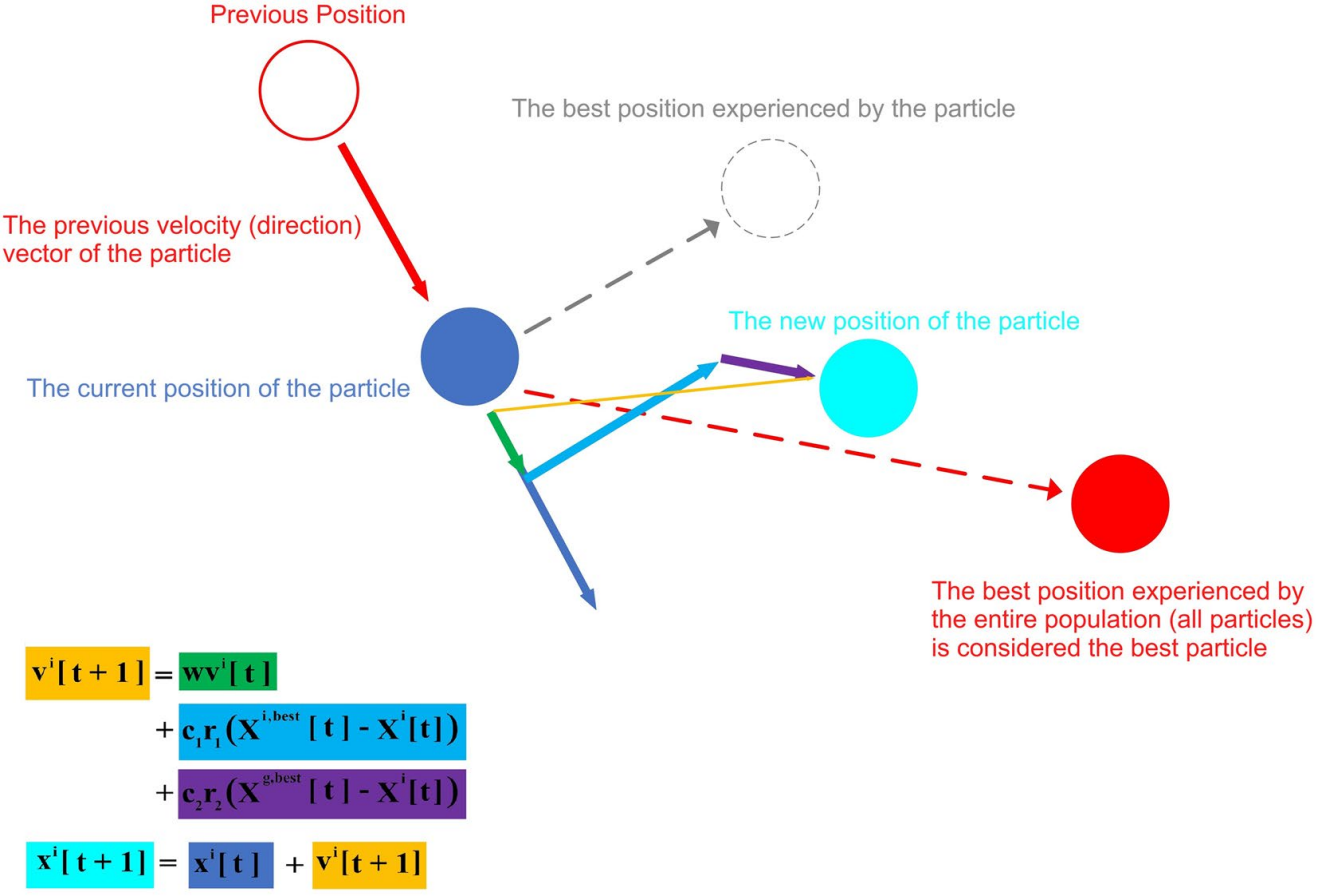
The behavior of a particle in PSO algorithm to reach the optimal value.

According to the calculations, the values of 0.7298 for the inertia coefficient and 1.4961 for the personal and collective learning coefficients have been calculated as the most optimal possible numbers. The equations describing the behavior of particles in the PSO algorithm are also schematically shown in Fig. 2.

**Figure 3:**
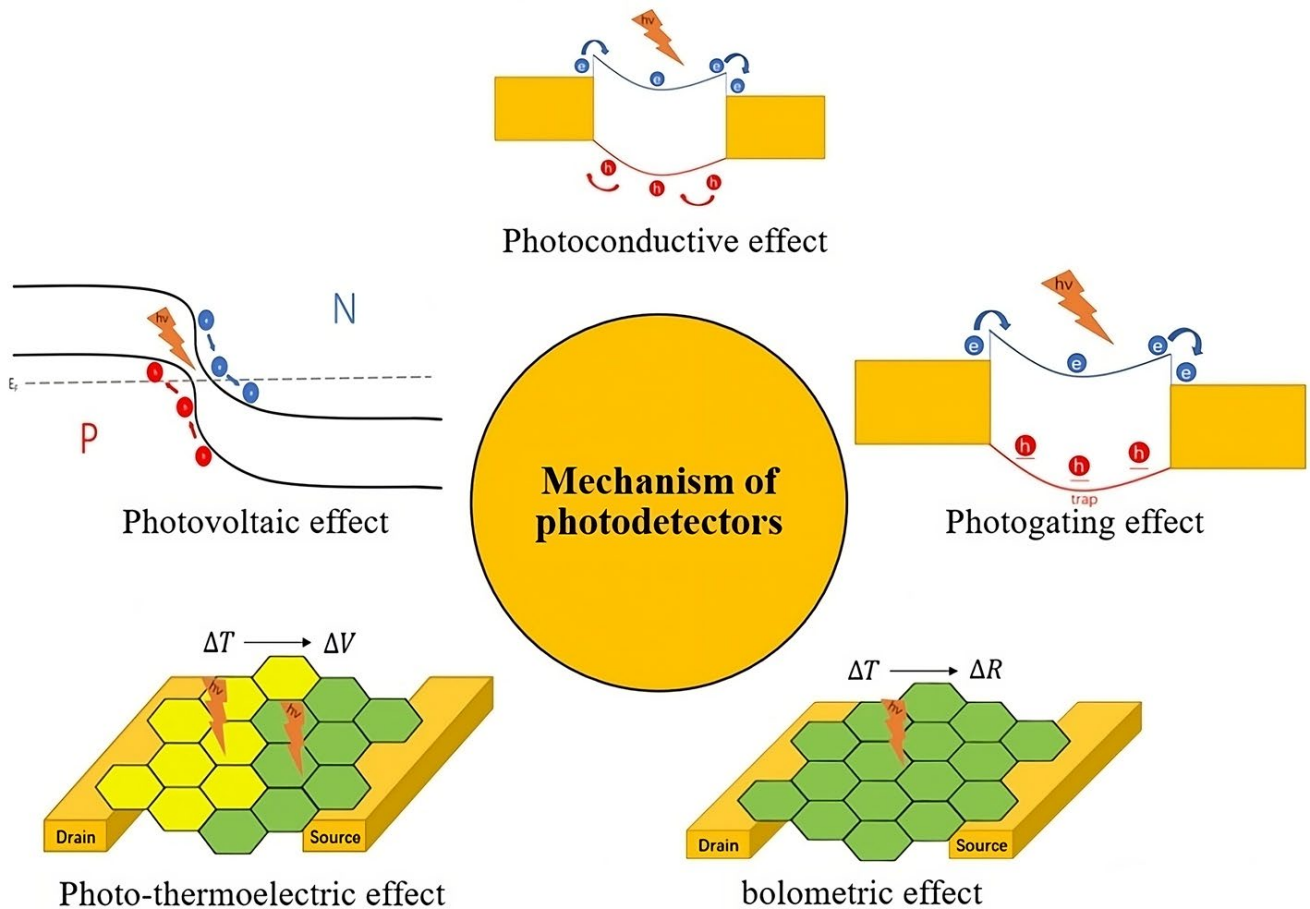
Mechanisms of photodetectors (Reference^79^, Fig. 2).

Photodetectors can generally be divided into two categories: photonic type (photovoltaic effect (PVE), photoconductive effect (PCE), photogating effect (PGE)) and thermal type (photothermoelectric effect (PTE), photobolometric effect (PBE))^79^. These detection mechanisms are shown in Fig. 3.

In the meantime, graphene, as a two-dimensional material used in this photodetector, performs detection operations mainly through three physical effects: bolometric^80^, photovoltaic^36,81,82^, and photothermoelectric^39,76,83–85^. The investigated device works under the photothermoelectric effect and does not need to apply external bias. Also, the photothermoelectric effect is dominant over the photovoltaic effect at zero bias. The photothermoelectric effect is based on the temperature gradient that is caused by the temperature difference between two positions of the semiconductor caused by non-uniform light radiation or absorption of light by materials^22^. The existence of this temperature difference between two points creates a voltage difference caused by the Seebeck effect. The induced photothermoelectric voltage is calculated through the following equation^86^:

4 $${\text{V}}_{\text{PTE}}\text{=}{\int }_{0}^{{\text{x}}_{0}}{\text{S}}\nabla {\text{T}}_{\text{c}}{\text{dx}}$$

The above formula shows that an asymmetric profile of the Seebeck coefficient can induce photothermoelectric voltage across the graphene sheet. When graphene comes in contact with metal, it becomes an impurity, and this changes the height of the Schottky barrier at the metal-graphene junction, thus changing the chemical potential and affecting the Seebeck coefficient and ultimately the PTE voltage. Here, TiN is considered as a metal (metal nitride), and graphene as a semiconductor (a semiconductor with a zero-band gap)^4^. To obtain the photothermoelectric voltage, carrier temperature characteristics, and Seebeck coefficient are needed. The carrier temperature profile across the graphene sheet is obtained by solving the heat transfer equation^87,88^:

5 $$-\kappa \frac{{\partial }^{2}{{\text{T}}}_{\text{c}}}{\partial {\text{x}}^{2}}\text{+}\gamma \text{C(}{\text{T}}_{\text{c}}-{\text{T}}_{0}\text{)=}{\text{A}}_{\text{G}}\text{I(x)}$$

where γ is the carrier cooling rate, C is the carrier heat capacity, ${\text{A}}_{\text{G}}$ is the effective optical absorption of graphene^69^ and I(x) is the excitation intensity profile of the waveguide mode. By finding the photothermoelectric voltage and dividing it by the total optical power input in the waveguide, the voltage responsivity is calculated. To calculate Eq. (5), we need to define the parameters involved, so, first, we start with the electrical conductivity (σ) of graphene, which is calculated as follows^89^:

6 $$\sigma \text{=}{\sigma }_{0}\left(\text{1} + \frac{{\mu }^{2}}{{\Delta }^{2}}\right)\text{, }{\sigma }_{0}= \text{5} \left(\frac{{\text{e}}^{2}}{\text{h}}\right)$$

where ${\upsigma }_{0}$ is the minimum conductivity, e is Electron charge, h is Planck's constant and $\Delta$ is the width of the charge-neutral region. Thermal conductivity (κ) is related to electrical conductivity (σ) through the Wiedmann-Franz law^89,90^:

7 $$\kappa \text{=}\frac{{\pi }^{2}{{\text{k}}}_{\text{B}}^{2}{\text{T}}}{{3}{\text{e}}^{2}}\sigma$$

Carrier cooling rate (γ) and related parameters are also expressed as follows^20,56,87,89^:

8 $$\gamma = \text{b} \left(\text{T} + \frac{{\text{T}}_{*}^{2}}{\text{T}}\right)$$

9 $$\text{b=2.2}\frac{{\text{g}}^{2}\vartheta {\text{k}}_{\text{B}}}{{\hbar}{\text{k}}_{\text{F}}{\text{l}}}\text{, }{\text{T}}_{*}\text{=}{\text{T}}_{\text{BG}}\sqrt{\text{0.43}{\text{k}}_{\text{F}}{\text{l}}}\text{,} \text{g} = \frac{\text{D}}{\sqrt{{2}\rho {\text{s}}^{2}}}$$

10 $$\vartheta = \frac{{2\mu }}{{\pi \hbar ^{2} \nu _{F}^{2} }},{\text{ }}k_{F} = \frac{\mu }{{\hbar \nu _{F} }},k_{F} l = \frac{{\pi \hbar \sigma }}{{e^{2} }},{\text{ }}T_{{BG}} = \frac{{s\hbar k_{F} }}{{k_{B} }}$$

g is the electron–phonon coupling constant, $\vartheta$ is the density of states, ${\text{k}}_{\text{F}}{\text{l}}$ is the mean free path, ${\text{k}}_{\text{F}}$ is the Fermi wave vector, ${\text{T}}_{\text{BG}}$ is the Bloch-Grüneisen temperature, D = 20 eV is the deformation potential constant, $\rho =\text{7.6}\times {1}{\text{0}}^{-7}{\text{Kg}}/{\text{m}}^{2}$ is the mass density of graphene, ${\text{v}}_{\text{F}}\text{=}{10}^{6}{\text{m}}/{\text{s}}$ is the Fermi velocity of hot carriers and $\text{s=2}\times {1}{\text{0}}^{4}{\text{m}}/{\text{s}}$ is the speed of longitudinal acoustic phonons. The heat capacity of the carrier and the cooling length of the carrier are also expressed as follows^4,20^:

11 $$\text{C} = \frac{{\pi }^{2}{{\text{k}}}_{\text{B}}^{2}{\text{T}}}{3}\vartheta , \xi =\sqrt{\frac{\kappa }{\gamma {\text{C}}}}$$

Now having the necessary parameters to solve the heat transfer equation, we can obtain the temperature profile of the carrier^4,89^:

12 $$\Delta \text{T} = {\text{T}}_{\text{c}}\text{(x)}-{\text{T}}_{0}\text{=}\frac{\xi {\text{sinh}}\text{(}\text{(}{\text{x}}_{0}-\left|{\text{x}}\right|\text{)/}\xi \text{)}}{{2}{\text{cosh}}\text{(}{\text{x}}_{0}\text{/}\xi \text{)}}\left(\frac{{\text{A}}_{\text{G}}{\text{I}}\text{(x)}}{\kappa }\right)$$

where ${\text{x}}_{0}$ is the distance from the peak of I(x) to the side electrode. To obtain the photothermoelectric voltage, the temperature of the carrier is multiplied by the Seebeck coefficient, which is obtained by the Mott formula^4,20,89,90^:

13 $$\text{S} = -\frac{{\pi }^{2}{{\text{k}}}_{\text{B}}^{2}{\text{T}}}{\text{3e}}\frac{1}{\sigma }\frac{{\text{d}}\sigma }{{\text{d}}\mu }$$

Also, the optical current is calculated by dividing the resulting PTE voltage by the resistance of the graphene sheet (${\text{R}}_{\text{G}}$)^4,89^:

14 $${\text{R}}_{\text{G}}\text{=}\frac{\text{w}}{{\text{L}}}{\sigma }^{-{1}}\text{(x)}$$

which w is the distance between the electrodes and L is the length of the photodetector. By dividing the photothermoelectric current by the total input power to the waveguide, the current response can be calculated. Since this device works at zero bias, it will have zero dark current, zero flicker, and zero-shot noise, and its NEP is determined only by Johnson noise^4^:

15 $$\text{NEP} = \frac{{\text{V}}_{\text{th}}}{{\text{R}}_{\text{V}}}{=}\frac{\sqrt{{4}{{\text{k}}_{\text{B}}{\text{T}}}{{\text{R}}_{\text{G}}}}}{{\text{R}}_{\text{V}}}$$

which ${\text{V}}_{\text{th}}$ is the variance of the thermal noise voltage per 1 Hz of the bandwidth, NEP is defined as the power of the input signal that results in an SNR ratio of one at an output bandwidth of 1 Hz, and ${\text{R}}_{\text{V}}$ represents the voltage responsivity. The following equation can be used to calculate the ${\text{R}}_{\text{V}}$^4^:

16 $${\text{R}}_{\text{V}}=\frac{{\text{V}}_{\text{PTE}}}{{\text{P}}_{\text{tot,in}}}$$

That ${\text{V}}_{\text{PTE}}$ is induced photothermoelectric voltage, ${\text{P}}_{\text{tot,in}}$ is the total input optical power in the waveguide. To calculate the current response, it is necessary to first define the photothermoelectric-induced current^4,69^:

17 $${\text{I}}_{\text{PTE}}\text{=}\frac{{\text{V}}_{\text{PTE}}}{{\text{R}}_{\text{G}}}$$

That ${\text{V}}_{\text{PTE}}$ is induced photothermoelectric voltage and ${\text{R}}_{\text{G}}$ is the graphene sheet resistance. Finally, the current responsivity (${\text{R}}_{\text{i}})$ can be calculated using the following equation^4^:

18 $${\text{R}}_{\text{i}}=\frac{{\text{I}}_{\text{PTE}}}{{\text{P}}_{\text{tot,in}}}$$

That ${\text{I}}_{\text{PTE}}$ is the photothermoelectric-induced current and ${\text{P}}_{\text{tot,in}}$ is the total input optical power in the waveguide. Also, the specific detectivity (D*) of the inverse NEP normalized to the square root of the active area is defined^21,26,57^:

19 $${\text{D}}^{*}\text{=}\frac{\sqrt{{\text{A}}\Delta {\text{f}}}}{\text{NEP}}$$

where A is the active (photosensitive) area, f is the electrical bandwidth, 1 Hz, and NEP is the noise equivalent power.

## Methods

The whole optimization procedure for the photodetector waveguide and techniques to enhance the efficient absorption of graphene under the plasmonic effect are extensively discussed in the references^4,69^. The present study employs the same values and evaluations as indicated in these references. The refractive index of TiN, ${\text{SiO}}_{2}$ and Si at the wavelength of 1550 nm is considered equal to $\text{2.54}+\text{7.84i}$, 1.5277 and 3.4777, respectively. But one of the important issues that needs to be addressed in the discussion of graphene-based photodetectors is the carrier transfer mechanism. Understanding the carrier transport mechanism in graphene is essential to utilizing its full potential in various electronic and optoelectronic applications. In graphene, a variety of transport mechanisms help move charge carriers (electrons or holes) through the material. These mechanisms can be categorized based on the nature of the movement and the dominant factors affecting carrier transport. Here are a few of the main types of transport mechanisms in graphene^91^:

- *Ballistic Transition:* This occurs when carriers move through a material without scattering. In pristine graphene samples with minimal defects or impurities, carriers can exhibit ballistic transport over relatively long distances due to the absence of backscattering. Ballistic transport is characterized by high carrier mobility and can dominate in high-quality graphene devices.
- *Saturation Transition:* Saturation transport refers to a regime where the carrier mobility saturates at high carrier densities or high electric fields. In graphene, the linear dispersion relation near the Dirac point leads to a characteristic behavior where carrier mobility reaches a saturation value as the carrier density increases. This saturation occurs due to phenomena such as screening effects, carrier-carrier interactions, and scattering processes becoming more pronounced at higher carrier densities.
- *Field-Effect Transition:* Field-effect transport refers to the modulation of carrier density and conductivity in graphene devices through the application of an external electric field. In graphene field-effect transistors (FETs), for example, the conductivity of graphene can be controlled by varying the gate voltage, which alters the carrier concentration in the material.
- *Thermal Transition:* Thermal transport in graphene refers to the movement of thermal energy through the material, primarily mediated by the motion of phonons. Graphene exhibits exceptional thermal conductivity due to its two-dimensional structure and the absence of Umklapp scattering at room temperature.
- *Diffusive Transition:* Diffusive transport occurs when carriers undergo random scattering events as they move through the material. In graphene, scattering mechanisms such as phonon scattering, impurity scattering, and edge scattering can cause carriers to diffuse through the lattice, leading to a net drift in the direction of the electric field. Diffusive transport is prevalent in graphene devices where scattering processes are significant, resulting in reduced carrier mobility compared to ballistic transport.
- *Intra-Band Transition:* Intra-band transfer involves the movement of carriers within the same energy band. In materials like graphene, which has a unique band structure with a Dirac cone at the Fermi level, carriers can move within the conduction band or the valence band. Intra-band transfer mechanisms include processes such as scattering by phonons, impurities, defects, or other carriers within the same band. These processes influence the mobility and conductivity of carriers in graphene.
- *Inter-band Transition:* Inter-band transfer involves the movement of carriers between different energy bands, typically between the conduction and valence bands. In graphene, inter-band transfer can occur when carriers are excited across the bandgap or when the Fermi level is shifted, such as in field-effect transistors. External perturbations, such as electric fields or optical excitation, can induce inter-band transitions.

Considering that graphene samples are unintentionally doped when they are placed on a substrate and lead to a change in chemical potential, This work models graphene, assuming a scattering time of 1 ps. The relationship $\tau \text{=}{1}/{2}\Gamma$ relates the dispersion time to the dispersion rate that $\Gamma$ is the scattering rate of graphene. On the other hand, an incident photon has an energy of ∼0.8 eV at $\lambda \text{=}\text{1550 nm}$. This photon induces an interband transition when absorbed by graphene since ${\hbar}\omega >{2}\mu$^69^. Also, the optical absorption of graphene is affected by the interband transition at the telecommunication wavelength for the chemical potential range used in this work^4^. On the other hand, surface optical conductivity (${\sigma }_{0}$) has been used to model graphene as a two-dimensional material, which is defined as follows:

20 $$\widetilde{\sigma }\text{(}\omega \text{,}\Gamma \text{,}\mu \text{,T)=}{\widetilde{\sigma }}_{\text{intra}}\text{(}\omega \text{,}\Gamma \text{,}\mu \text{,T)+}{\widetilde{\sigma }}_{\text{inter}}\text{(}\omega \text{,}\Gamma \text{,}\mu \text{,T)}$$

21 $${\widetilde{\sigma }}_{\text{intra}}\text{(}\omega \text{,}\Gamma \text{,}\mu \text{,T)=}\frac{\text{-j}{\text{e}}^{2}}{\pi {\hbar}^{2}\text{(}\omega + \text{j2} \Gamma \text{)}}\times{ \int\limits_{0}^{\infty } {{\text{E}}\left( {\frac{{\partial {\text{f(E)}}}}{{\partial {\text{E}}}} - \frac{{\partial {\text{f( - E)}}}}{{\partial {\text{E}}}}} \right){\text{dE}}} }$$

22 $${\widetilde{\sigma }}_{\text{inter}}\text{(}\omega \text{,}\Gamma \text{,}\mu \text{,T)=}\frac{\text{-j}{\text{e}}^{2}\text{(}\omega + \text{j2} \Gamma \text{)}}{\pi {\hbar}^{2}}\times{\int }_{0}^{\infty}\frac{\text{f(-E)-f(E)}}{\text{(}\omega + \text{j2} \Gamma {)}^{2} {-4(E/\hbar}{)}^{2}}{\text{dE}}$$

23 $$\text{f(E)=(}{\text{e}}^{\text{(E-}\mu \text{)/}{\text{k}}_{\text{B}}{\text{T}}}+ \text{1} {)}^{-1}$$

${\widetilde{\sigma }}_{\text{intra}}$ and ${\widetilde{\sigma }}_{\text{inter}}$ account for the surface optical conductivity due to intraband and interband transitions, respectively. $\upomega$ is the angular frequency of incident photons, T is the operation temperature, e is the electron charge, ${\hslash }$ is the reduced Planck constant, $\text{f}\left(\text{E}\right)$ is the Fermi–Dirac distribution, and kB is the Boltzmann constant. The surface electric permittivity ($\widetilde{\in }$) and the surface electric susceptibility ($\widetilde{\upchi }$) are related to $\widetilde{\upsigma }$ through the following relation ^92^:

24 $$\widetilde{\varepsilon }\text{=}{\varepsilon }_{0}\widetilde{\chi }+ \text{j} \frac{\widetilde{\sigma }}{\omega }$$

In an photodetector, various types of noise mechanisms can be caused by various sources such as external factors or inherent characteristics of the device. Here are the main types of noise mechanisms in photodetectors^93–95^:

- *Shot noise:* Arises from the statistical nature of the arrival of photons at the detector. It is inherent in any photoelectric process and is proportional to the square root of the signal.
- *Thermal noise (Johnson-Nyquist noise):* Caused by the random motion of charge carriers (electrons and holes) due to thermal energy. It is present in all resistive elements in the circuit, including the photodetector.
- *Dark current noise:* Results from the random generation and recombination of electron–hole pairs within the photodetector even in the absence of light. Dark current is typically due to thermal effects or defects in the detector material.
- *Flicker (1/f) noise:* Occurs at low frequencies and is often attributed to fluctuations in the number of trapping and detrapping events in the detector material.
- *Excess noise:* Additional noise beyond shot noise, often observed in avalanche photodetectors due to the impact ionization process.
- *Amplifier noise:* Noise contributed by the amplifier used to read the signal from the photodetector, including voltage and current noise from the amplifier circuitry.

## Results and discussion

We acknowledge that the data analysis was conducted using MATLAB (software), R2023b (version), Core i7-8750H (CPU), 12 GB (RAM), and 3 GB of NVIDIA Geforce GTX 1060 (GPU) for writing and preparing this manuscript. In this section, the results of the implementation of the NEP as the objective function in the PSO algorithm are presented. Although the main goal of this article is to optimize the NEP, due to the direct connection of specific detectivity and response parameters with the variables in the NEP parameter, we have seen a significant improvement in these parameters as well. First, it is necessary to rewrite the NEP relation. In the article^4^, Eq. (15) is considered for the NEP, which depends only on two parameters $\mu$ and $\Delta$. In this work, in addition to the fact that $\Delta$ is considered as a continuous interval like $\mu$, the NEP, it is rewritten based on four other parameters that can be variable. With this work, the NEP becomes dependent on six independent variables in total, each of these variables is also defined in a permissible range and in a way forms a six-dimensional search space that the PSO algorithm is defined in this space and after the global searches it performs, it obtains the most optimal possible value for the NEP.

25 $$NEP = \frac{{\sqrt {4 \times k_{B} T\left( {\frac{w}{L}\sigma^{ - 1} } \right)} }}{{\frac{{\int_{0}^{{x_{0} }} {S\nabla T_{c} dx} }}{{P_{tot,in} }}}} \, = \frac{{\left( {\sqrt {4 \times k_{B} T\left( {\frac{w}{L}\sigma^{ - 1} } \right)} } \right) \times P_{tot,in} }}{{ - \frac{{S\xi A_{G} I(0)}}{2 \times \kappa } \times \tanh \left( {\frac{{x_{0} }}{\xi }} \right)}} = \frac{{\left( {\sqrt {4 \times k_{B} T\left( {\frac{w}{L}\sigma^{ - 1} } \right)} } \right) \times P_{tot,in} }}{{ - \frac{0.0225 \times S\xi }{\kappa } \times \tanh \left( {\frac{{x_{0} }}{\xi }} \right)}}.$$

The above relationship shows that the NEP depends on the variables w, L, T, $\mu$, $\Delta$, and ${\text{x}}_{0}$. It should be noted that the selected intervals for the parameters have been chosen in such a way that these conditions can be provided in practice. For example, the chemical potential of graphene can be adjusted using the thermal annealing method. To calculate the voltage responsivity, current responsivity, and specific detectivity, the relationships and explanations mentioned have been used. Also, the units of each of the mentioned variables are $\text{w(}\mu \text{m)}$,$\text{L(}\mu \text{m)}$, $\text{T(K)}$, $\mu \text{(eV)}$,$\Delta \text{(eV)}$, and ${\text{x}}_{0}\text{(}\mu \text{m)}$ respectively. The equivalent power unit of noise is also ${\text{pW}}/\sqrt{\text{Hz}}$. In the following, the optimal values of these parameters for different numerical ranges defined for the mentioned variables were calculated and compared with each other and also with the main reference. In order to have a better understanding of how the PSO algorithm works, two pseudo-codes are provided that can help a lot to understand how this algorithm works. The first pseudo-code shows how to define the objective function (NEP) and the second pseudo-code completely shows the PSO algorithm, how to add the objective function to the algorithm, define the constraints, and customize this algorithm for the present work. As mentioned, Algorithm 1 shows how to define the objective function. First, a handle function named NEP has been defined. This work, in addition to increasing the readability of the written code, helps to increase the flexibility and speed of processing the written code. In the following, the order of the variables is determined according to the fact that an interval is considered for each of them in the main body of PSO. Finally, considering that the necessary settings have been made in the previous steps, the desired objective function (Eq. (25)) is implemented as code. It should be noted that the written code is saved separately and placed in a file together with the original code, which includes Algorithm 2. Pay Attention To the requirement for the correct execution of the written program depends on the fact that all the written programs are in one file.

**Algorithm 1:**
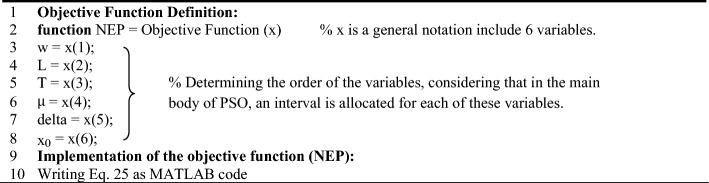
Pseudo code to define the objective function.

Next, it is time to write the desired main program. This program includes adding the objective function written in Algorithm 1, defining the parameters used by the PSO algorithm, implementing the necessary initial definitions, and then writing the main body of the PSO algorithm, which has finally led to the presentation of results in the form of figures and numerical data. First, the objective function is added to this algorithm. The number of variables and the target interval are also defined for each of these variables. In the second step, it is time to define the parameters of the PSO algorithm itself. In this section, there are two important points that are worth mentioning. The first point is about the inertia-weight damping ratio. The "Inertia Weight Damping Ratio" in the PSO algorithm is a parameter used to gradually decrease the impact of the inertia weight on particle velocities during optimization. It is typically a value between 0 and 1 that is multiplied by the current inertia weight at each iteration. This damping helps balance exploration and exploitation in the algorithm, improving convergence towards an optimal solution. Unlike the "inertia weight ratio," which is a constant initial value for the inertia weight, the inertia weight damping ratio is applied multiplicatively to the current inertia weight at each iteration to decrease its value over time and adjust the inertia weight dynamically throughout the optimization process. The second point is regarding the use of two different methods to calculate the inertia coefficient, the personal learning coefficient and the collective learning coefficient. Coefficients 1, as shown in Algorithm 2, are conventional coefficients used in the PSO algorithm. Meanwhile, coefficients 2 are known as contraction coefficients. These coefficients are used in practice to improve the convergence and stability of the algorithm, adjust the velocity update equation, and especially to balance the best personal position of the particle, the best global position and its current velocity. In the present work, both coefficients were investigated, and the results of contraction coefficients were better than conventional coefficients, so these coefficients were selected. One of the most important parts of the PSO algorithm is how to define and apply the speed limit, for which existing and conventional relationships are used. The next step is to set the initial position and speed of the particles in the search space. After going through these steps, it is time to write the main body of the PSO algorithm. This algorithm should search for and calculate the number of repetitions defined in the previous steps, so a loop is defined with a condition equal to the number of repetitions. The operation of search, evaluation, and calculation of values should be done for each particle of the population. of the population that this parameter was also defined previously, so another loop is defined with the condition equal to the number of members of the population. Now, after completing these steps, we apply the key relationships of the pso algorithm along with the necessary conditions to the main body of the code. The key point in this section is the correct application of the mirror effect. During the optimization process, particles can sometimes move beyond the predefined boundaries of the search space. Handling these out-of-bounds particles is critical to algorithm performance. The velocity mirror effect is one strategy used to manage particles that exceed boundaries. When a particle crosses the boundary, this effect reflects both the particle's position and its velocity. Essentially, the particle "returns" to the search space and its velocity is reversed or reflected. The velocity mirror effect helps maintain crowd diversity and prevents particle stagnation at boundaries. This ensures that the particles remain in the search space and allows the PSO algorithm to continue to explore potential solutions efficiently. In the following, the value of the objective function is calculated. After calculating the value of the objective function, it is time to update the position and velocity values of the particles. At this stage, if the value of the objective function of the desired particle is expressed here as the cost function (in general, in optimization discussions, if the goal is minimization, the term cost/error function is used, and if the goal is maximization, the term profit/fitness function is used), is better than the best possible value for it that is already available, this value will replace the previous one, and further, this value is also compared with the best overall value that is already available, and if the value of the particle's objective function If the target is better than the total value, in this case, the total value is also updated and equal to the value of the target function, which will be used for the next iterations. If the first-mentioned condition is false, the algorithm will immediately repeat all calculations for the next particle. This process was applied in each repetition and for each particle and the values were calculated and compared according to the explanations given. Finally, the output results are displayed as figures and numerical data by obtaining the best possible value for the objective function.

**Algorithm 2:**
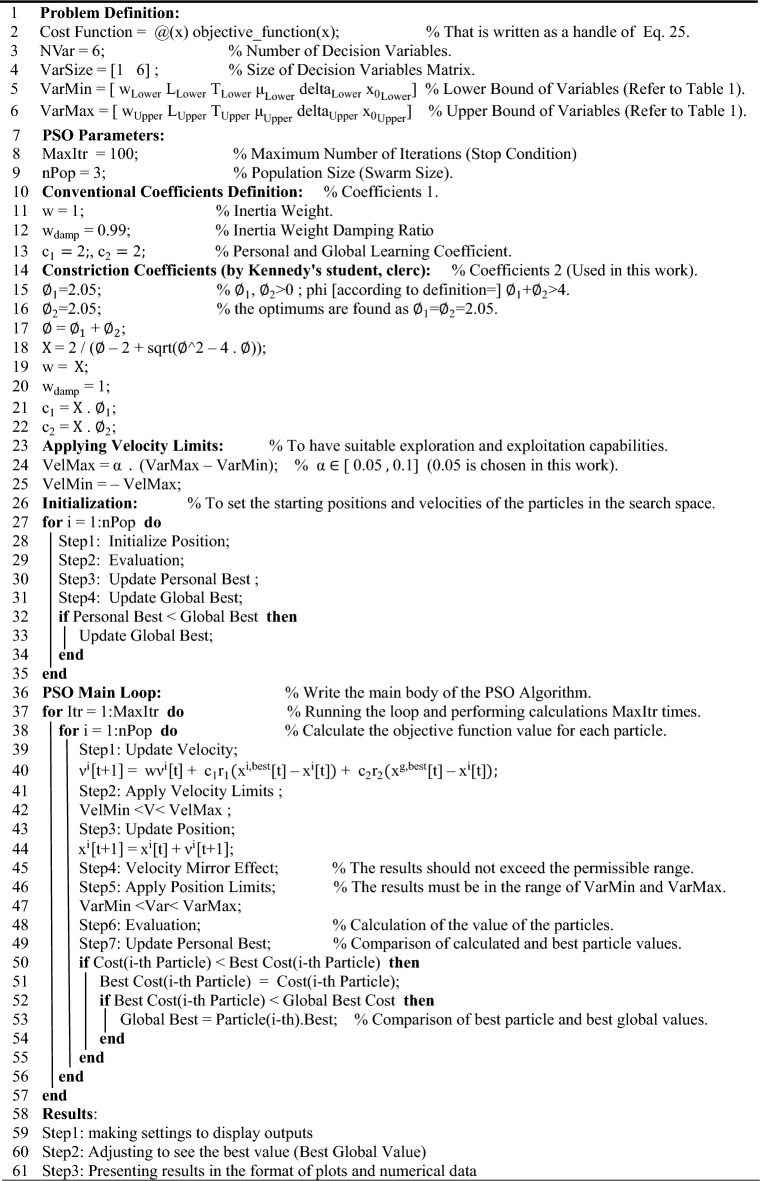
Pseudo code for the proposed PSO algorithm.

### Optimal values of variables

**Figure 4:**
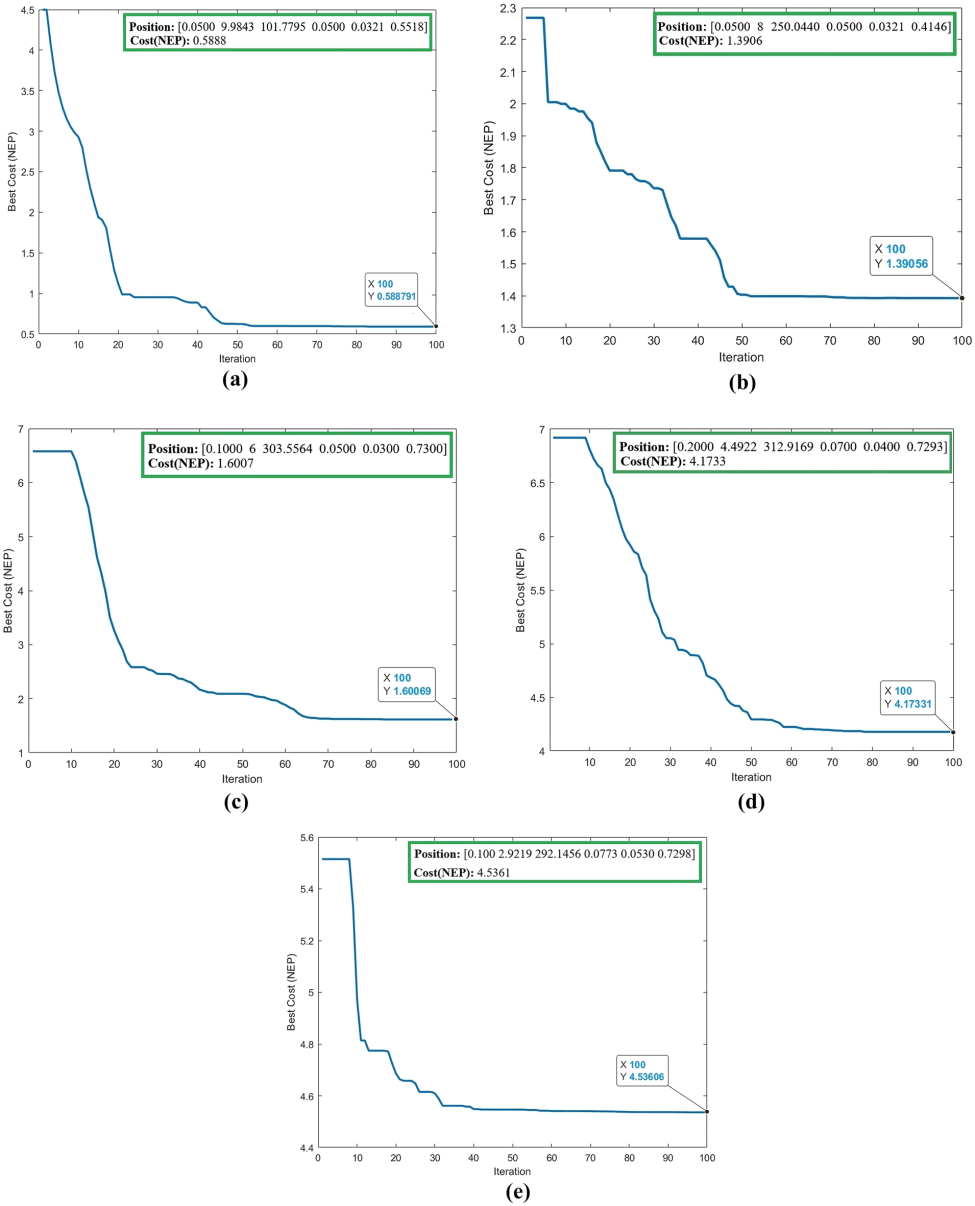
Figures (**a**–**e**) correspond to cases 1 to 5 in Table 1, respectively; Graphs provided by PSO for NEP.

In this section, 5 different cases are considered for the 6 mentioned variables. In each of these situations, special conditions have been defined for the variables, so that the PSO algorithm, after performing the calculations, obtains the optimal value of these variables for each of the situations. and then, it calculates the lowest possible value for the NEP parameter. The obtained results are shown in Table 1. Also, the calculation process by the PSO algorithm is shown in Fig. 4.

**Table 1:** The results of NEP optimization using the PSO algorithm for different cases.

Case	Defined intervals	Results provided by PSO	NEP
1	$\text{w} = \left[\text{0.05 0.5}\right]$, $\text{L} = \left[\text{3 10}\right]$ $\text{T} = \left[\text{100 500}\right]$, $\mu \text{=}\left[\text{0.05 0.1}\right]$ $\Delta \text{=}\left[\text{0.01 0.09}\right]$,${\text{x}}_{0}\text{=}\left[\text{0.28 0.73}\right]$	$\text{w=0.05 }\mu {\text{m}}$, $\text{L=9.9843 }\mu {\text{m}}$ $\text{T=101.7795 K}$, $\mu \text{=0.05 eV}$ $\Delta \text{=0.0321 eV}$,${\text{x}}_{0}\text{=0.5518 }\mu {\text{m}}$	$\text{0.}\text{5888 }{\text{p}}{\text{W}}/\sqrt{\text{Hz}}$
2	$\text{w} = \left[\text{0.05 0.5}\right]$, $\text{L} = \left[\text{3 8}\right]$ $\text{T} = \left[\text{250 350}\right]$, $\mu \text{=}\left[\text{0.05 0.1}\right]$ $\Delta \text{=}\left[\text{0.02 0.08}\right]$,${\text{x}}_{0}\text{=}\left[\text{0.28 0.73}\right]$	$\text{w=0.05 }\mu {\text{m}}$, $\text{L=8 }\mu {\text{m}}$ $\text{T=250.044 K}$, $\mu \text{=0.05 eV}$ $\Delta \text{=0.0321 eV}$,${\text{x}}_{0}\text{=0.4146 }\mu {\text{m}}$	$\text{1.3906 }{\text{pW}}/\sqrt{\text{Hz}}$
3	$\text{w} = \left[\text{0.1 0.5}\right]$, $\text{L} = \left[\text{3 6}\right]$ $\text{T} = \left[{2}{\text{7}}\text{0 330}\right]$, $\mu \text{=}\left[\text{0.05 0.1}\right]$ $\Delta \text{=}\left[\text{0.03 0.07}\right]$,${\text{x}}_{0}\text{=}\left[\text{0.33 0.73}\right]$	$\text{w=0.1 }\mu {\text{m}}$, $\text{L=6 }\mu {\text{m}}$ $\text{T=303.5564 K}$, $\mu \text{=0.05 eV}$ $\Delta \text{=0.03 eV}$,${\text{x}}_{0}\text{=0.73 }\mu {\text{m}}$	$\text{1.6007 }{\text{pW}}/\sqrt{\text{Hz}}$
4	$\text{w} = \left[\text{0.2 0.5}\right]$, $\text{L} = \left[\text{3 4.5}\right]$ $\text{T} = \left[\text{280 320}\right]$, $\mu \text{=}\left[\text{0.07 0.1}\right]$ $\Delta \text{=}\left[\text{0.04 0.06}\right]$,${\text{x}}_{0}\text{=}\left[\text{0.43 0.73}\right]$	$\text{w=0.2 }\mu {\text{m}}$, $\text{L=4.4922 }\mu {\text{m}}$ $\text{T=312.9196 K}$, $\mu \text{=0.07 eV}$ $\Delta \text{=0.04 eV}$,${\text{x}}_{0}\text{=0.7293 }\mu {\text{m}}$	$\text{4.1733 }{\text{pW}}/\sqrt{\text{Hz}}$
5	$\text{w} = \left[\text{0.1 0.5}\right]$, $\text{L} = \left[\text{2 3}\right]$ $\text{T} = \left[\text{290 310}\right]$, $\mu \text{=}\left[\text{0.07 0.1}\right]$ $\Delta \text{=}\left[\text{0.045 0.055}\right]$,${ \, {\text{x}}}_{0}\text{=}\left[\text{0.63 0.73}\right]$	$\text{w=0.1 }\mu {\text{m}}$, $\text{L=2.9219 }\mu {\text{m}}$ $\text{T=292.1456 K}$, $\mu \text{=0.0773 eV}$ $\Delta \text{=0.053 eV}$,${\text{x}}_{0}\text{=0.7298 }\mu {\text{m}}$	$\text{4.5361 }{\text{pW}}/\sqrt{\text{Hz}}$

**Figure 5:**
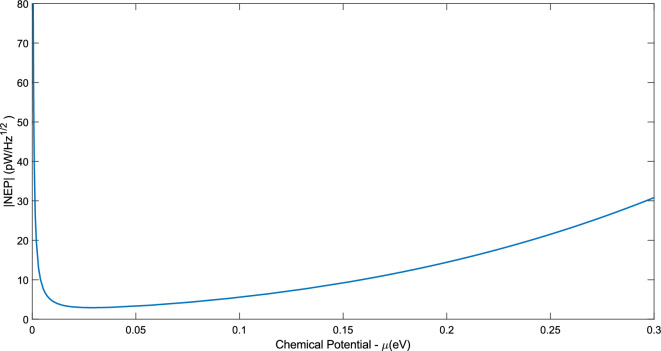
NEP as a function of chemical potential.

In Fig. 5, according to the optimal values selected in Table 1, the noise equivalent power curve is shown. To draw this curve, all the selected values (Case 5 from Table 1) were put in the equation related to this parameter. Also, since if all the variables were considered as a constant number, the final answer would also be a constant value, in order to get a curve for this parameter and to examine its behavior, the chemical potential variable is a The interval was considered and the desired curve was drawn.

### Optimal values of $${\text{R}}_{\text{v}},$$$${\text{R}}_{\text{i}},\boldsymbol{ }{\text{D}}^{*}$$ and comparison with the reference article

In this section, for each of the above 5 cases, in addition to the NEP, voltage responsivity, current responsivity, and specific detectivity are also calculated and compared with the values of reference article 1, which are shown in Table 2. Case 5 is chosen as the best trade-off between these cases because according to Table 1, it provides operational values for the variables.

**Table 2:** The results of ${\text{R}}_{\text{v}}$,${\text{R}}_{\text{i}}$ and ${\text{D}}^{*}$ optimization using the PSO algorithm for different cases.

Case	Voltage responsivity	Current responsivity	Specific detectivity
1	$\text{350.3893 }{\text{V}}/{\text{W}}$	$\text{46.3517 }{\text{A}}/{\text{W}}$	$\text{36.397}\times {10}^{7}\text{ Jones}$
2	$\text{259.5702 }{\text{V}}/{\text{W}}$	$\text{27.5132 }{\text{A}}/{\text{W}}$	$\text{13.794}\times {10}^{7}\text{ Jones}$
3	$\text{386.3485 }{\text{V}}/{\text{W}}$	$\text{16.9324 }{\text{A}}/{\text{W}}$	$\text{10.378}\times {10}^{7}\text{ Jones}$
4	$\text{237.0949 }{\text{V}}/{\text{W}}$	$\text{4.1831} {\text{A}}/{\text{W}}$	$\text{3.444}\times {10}^{7}\text{ Jones}$
5	$\text{210.6215 }{\text{V}}/{\text{W}}$	$\text{3.7213 }{\text{A}}/{\text{W}}$	$\text{2.556}\times {10}^{7}\text{ Jones}$
Ref. ^4^	$\text{52.4519 }{\text{V}}/{\text{W}}$	$\text{0.7717 }{\text{A}}/{\text{W}}$	$\text{0.627}\times {10}^{7}\text{ Jones}$

**Figure 6:**
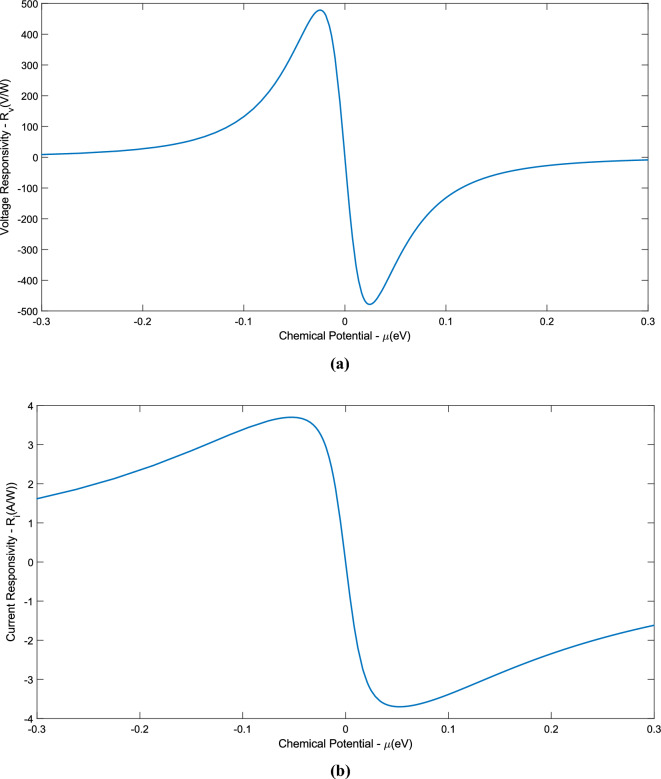
Voltage and current responsivity of the photodetector as a function of the chemical potential.

Also in Fig. 6, according to the optimal values selected in Table 1, the response curves of voltage and current are shown. To draw these curves, all the selected values (Case 5 from Table 1) were placed in the equation related to these parameters. And like plotting the noise equivalent power curve, since if all the variables are considered as a constant number, the final answer will also be a constant value, to obtain the curve for these parameters and examine their behavior, the chemical potential variable is The form of a continuous interval from $\text{-0.3}$ eV to 0.3 eV was considered.

### Comparison of performance metrics and features of photodetectors

Table 3 provides a summary of the performance metrics and characteristics of some of the recently reported peer-to-peer photodetectors.

**Table 3:** The performance metrics and characteristics of some recent photodetector.

Ref	Mechanism	Wavelength (nm)	Detectivity (Jones)	Applied bias (V)	Responsivity (A/W or V/W)	NEP (${\text{pW}}/\sqrt{\text{Hz}}$)	Device length ($\mu {\text{m}}$)	Year
^4^	PTE	1550	$\text{0.627}\times {10}^{7}$	0 V	$\text{52.45}{19} \, {\text{V}}/{\text{W}}$	20.22	3.5	2021
^16^	BPVE	LWIR	–	0 V	6.3 V/W	1600	–	2023
^21^	RTP	405–1050	$\text{7.2}\times {10}^{11}$	0 V	1948 mA/W	0.001–0.1	–	2022
^22^	PTE	1550	–	0 V	2.81 V/W	2000	60	2023
^26^	PTE + PVE	1310	$\text{0.28}\times {10}^{7}$	− 1 V	2.95 mA/W	–	–	2023
^32^	PTE	1550	–	0 V	0.6 A/W	22	30	2022
^38^	PTE	N/A	–	0 V	–	1000	44	2022
^39^	PTE	MWIR to LWIR	$\text{6.8}\times {10}^{7}$	N/A	2.5 V/W	–	1000	2022
^56^	PTE	1550	–	0 V	90 V/W	-	6	2021
^57^	PBE	0.22 THz	$\text{0.05}\times {10}^{9}$	1 V	805 mA/W	2740	100	2022
^58^	PBE	1550	–	1.9 V	603.92 mA/W	–	30	2022
^59^	–	365	$\text{1.56}\times {10}^{12}$	5 V	0.82 A/W	0.0658	50	2022
^60^	PBE	1300–1400	–	0.5 V	0.67 A/W	–	5	2020
^85^	PTE	0.105 THz	–	0 V	1.54 A/W	19.8	–	2023
^96^	IPE	1550	–	− 0.66 V	133 mA/W	0.5	–	2021
Pro	PTE	1550	$\text{2.556}\times {10}^{7}$	0 V	210.6215 V/W	4.536	2.9219	Pro

## Conclusion

In this work, a graphene-based plasmonic photodetector that operates at the telecom wavelength of 1.55 $\mu {\text{m}}$ has been optimized using the PSO algorithm. Accurate selection of influential variables in each parameter and obtaining the most optimal possible value for them by considering all conditions will create an efficient device. The importance of optimization is especially important in the discussion of manufacturing, because it saves money and time, and the optimized device will show the best performance. So here, first, the key parameters of the photodetector, i.e. NEP, responsivity, specific detectivity, and device length were selected as key parameters. In the following, considering various conditions for the variables, the optimal values for each of these parameters were calculated by the PSO algorithm. The NEP value for this device was obtained $\text{4.5361 }{\text{pW}}/\sqrt{\text{Hz}}$, which shows a 77.6% reduction compared to the previous work. The responsivity and specific detectivity of this device were obtained 210.6215 V/W and $\text{2.556}\times {10}^{7}$ Jones, respectively, which has shown an increase of more than 4 times for both parameters compared to the previous work. The length of the photodetector has been reduced by more than 16.5% compared to the previous work and is equal to 2.9219 $\mu {\text{m}}$, which is one of the ultra-compact photodetectors ever reported. These optimal values are obtained due to the adjustment of the variables by the PSO algorithm. The presented photodetector operates under zero bias, relying on the PTE effect which has a significant effect in reducing the amount of noise. Also, this device can be a suitable candidate for on-chip photodetector and is compatible with CMOS. In addition, the proposed photodetector has high resolution and accuracy due to its high responsiveness and has a high potential for use in medical imaging applications. Also, this type of device can be very useful in integration with integrated systems due to their very short length. On the other hand, this photodetector, having a very low noise equivalent power, can be used in chromatographic devices, which require low noise for the analysis and separation of chemicals with high sensitivity. This photodetector has a suitable special detection, which can make it a suitable candidate in the fields of cell biology and for the detection of living cells. Finally, this photodetector has been designed and analyzed in the telecommunication wavelength, so it is a very suitable option for high-speed telecommunication applications.

### Future trends

Examining and understanding future trends in this field will help students and researchers to keep up with scientific changes and innovations and provide higher quality and more effective research. Therefore, several important trends that can be expressed in relation to the present work are mentioned below.

- *Integration with CMOS Technology:* Future photodetectors will increasingly integrate with complementary metal–oxide–semiconductor (CMOS) technology, leveraging materials like titanium nitride (TiN) for compatibility and enhanced performance.
- *Graphene and Two-Dimensional Materials:* The use of graphene and other two-dimensional materials (such as Transition Metal Dichalcogenides, Phosphorene, Hexagonal Boron Nitride, and MXenes) will expand due to their unique properties like high carrier mobility, broad spectral response, and excellent mechanical strength.
- *Plasmonic Effects:* Utilizing plasmonic effects to overcome size limitations and enhance optical absorption will be a key trend. This involves engineering plasmonic environments around two-dimensional materials to optimize light-matter interactions.
- *Metaheuristic Optimization Algorithms:* The application of metaheuristic optimization algorithms, such as Particle Swarm Optimization (PSO), Genetic Algorithms (GA), and Ant Colony Optimization (ACO), will grow. These algorithms will optimize device parameters for enhanced performance, such as responsivity, noise equivalent power (NEP), and specific detectivity.
- *Zero-Bias and Low-Power Operation:* Photodetectors operating at zero bias and with low power consumption will become more prevalent, driven by the demand for energy-efficient devices with minimal noise and high sensitivity.
- *Scalable and Cost-Effective Fabrication:* There will be a focus on simplifying device structures and using materials that are compatible with existing semiconductor fabrication processes to reduce production costs and enhance scalability.

By understanding these trends, researchers and developers can align their efforts with the evolving landscape, ensuring that their work remains relevant and impactful in the field of photodetectors and related optoelectronic devices.

## Data Availability

The data that support the findings of this study are available from the corresponding author upon reasonable request.

## Abbreviations

ACO: Ant colony optimization
BOX: Bottom oxide
BPD: Balanced photodetector
CMOS: Complementary metal–oxide–semiconductor
CVD: Chemical vapor deposition
FDTD: Finite-difference time-domain
FET: Field-effect transistors
GA: Genetic algorithm
GaAs: Gallium arsenide
GPD: Graphene photodetectors
hBN: Hexagonal boron nitride
InP: Indium phosphide
MOGA: Multi-objective genetic algorithm
NEP: Noise equivalent power
PBE: Photobolometric effect
PCE: Photoconductive effect
PD: Photodetector
PDCR: Photocurrent-to-dark current ratio
PGE: Photogating effect
PIC: Photonic integrated circuit
PSO: Particle swarm optimization
PTE: Photothermoelectric effect
PVE: Photovoltaic effect
SLG: Single layer graphene
SNR: Signal-to-noise ratio
SPP: Surface plasmon polariton
TCR: Temperature coefficient of resistance
TiN: Titanium nitride
TM: Transverse magnitude
TMD: Transition metal dichalcogenide
UV: Ultraviolet

## Symbols

A: The active area
C: Carrier heat capacity
D: The deformation potential constant
e: The electron charge
g: The electron–phonon coupling constant
h: The Planck’s constant
$\hbar$: The reduced Planck constant
I: The excitation intensity profile of the waveguide mode
L: The length of the photodetector
S: The Seebeck coefficient
s: The speed of longitudinal acoustic phonons
T: Temperature
w: The inertia coefficient/The distance between the electrodes

## Superscripts

${\text{D}}^{*}$: The specific detectivity
${\text{v}}^{\text{i}}$: The speed of the ${\text{i}}^{\text{th}}$ particle
${\text{x}}^{\text{g}}$: The collective best memory
${\text{x}}^{\text{i}}$: The personal best memory
$\widetilde{\in }$: The surface electric permittivity
$\widetilde{\chi }$: The surface electric susceptibility

## Subscripts

${\text{A}}_{\text{G}}$: The effective optical absorption of graphene
${\text{c}}_{1}$: The personal learning coefficient
${\text{c}}_{2}$: The collective learning coefficient
${\text{I}}_{\text{PTE}}$: The photothermoelectric-induced current
${\text{k}}_{\text{B}}$: The Boltzmann constant
${\text{k}}_{\text{F}}$: The Fermi wave vector
${\text{k}}_{\text{F}}$ l: The mean free path
${\text{P}}_{\text{tot,in}}$: The total input power to the waveguide
${\text{r}}_{1}$ and${\text{r}}_{2}$: The random numbers
${\text{R}}_{\text{G}}$: The graphene resistance
${\text{R}}_{\text{i}}$: The current responsivity
${\text{R}}_{\text{v}}$: The voltage responsivity
${\text{T}}_{*}$: The crossover temperature
${\text{T}}_{0}$: The lattice temperature
${\text{T}}_{\text{BG}}$: The Bloch-Grüneisen temperature
${\text{T}}_{\text{c}}$: The carrier temperature
${\text{v}}_{\text{F}}$: The Fermi velocity of hot carriers
${\text{V}}_{\text{PTE}}$: The induced photothermoelectric voltage
${\text{V}}_{\text{th}}$: The variance of the thermal noise voltage
${\text{x}}_{0}$: The distance from the peak of excitation intensity to the side electrode
${\widetilde{\sigma }}_{\text{intra}}$: The intraband transition surface optical conductivity
${\widetilde{\sigma }}_{\text{inter}}$: The interband transition surface optical conductivity

## Greek symbols

$\Gamma$: The scattering rate
$\gamma$: The carrier cooling rate
$$\Delta$$: Difference
$\vartheta$: The density of state
$\kappa$: The thermal conductivity
$\mu$: The chemical potential
$\xi$: The cooling length of the carrier
$\rho$: The mass density of graphene
$\sigma$: The electrical conductivity
${\sigma }_{0}$: The minimum conductivity
$$\nabla$$: The gradient
$\partial$: The partial derivative

## References

[1] Li L-L (2024). Silicon-based optoelectronic heterogeneous integration for optical interconnection. Chin. Phys. B.

[2] Siew SY (2021). Review of silicon photonics technology and platform development. J. Light. Technol..

[3] Huang Y-C (2021). Silicon-based photodetector for infrared telecommunication applications. IEEE Photonics J..

[4] AlAloul M, Rasras M (2021). Plasmon-enhanced graphene photodetector with CMOS-compatible titanium nitride. J. Opt. Soc. Am. B.

[5] Wang Z, Cai Y, Jiang H, Tian Z, Di Z (2024). Graphene-based silicon photonic devices for optical interconnects. Adv. Funct. Mater..

[6] Chen X, Lin J, Wang K (2023). A review of silicon-based integrated optical switches. Laser Photonics Rev..

[7] Zhang J, Wang Y, Li D, Sun Y, Jiang L (2022). Engineering surface plasmons in metal/nonmetal structures for highly desirable plasmonic photodetectors. ACS Mater. Lett..

[8] Dorodnyy A (2018). Plasmonic photodetectors. IEEE J. Sel. Top. Quantum Electron..

[9] De Nicola F (2020). Graphene plasmonic fractal metamaterials for broadband photodetectors. Sci. Rep..

[10] Alaloul M (2023). Mid-wave infrared graphene photodetectors with high responsivity for on-chip gas sensors. IEEE Sens. J..

[11] Kim YJ (2023). Flexible TiN/Ge photodetectors with enhanced responsivity *via* localized surface plasmon resonance and strain modulation. J. Mater. Chem. C.

[12] Kaur HJ (2023). Investigation of germanium-based plasmonic-photo-detector improved by dual-absorption method using titanium nitride. J. Opt..

[13] Ding Y (2020). Ultra-compact integrated graphene plasmonic photodetector with bandwidth above 110 GHz. Nanophotonics.

[14] Jian J (2023). High-speed compact plasmonic-PdSe_2_ waveguide-integrated photodetector. ACS Photon..

[15] Liu X (2023). Plasmon resonance-enhanced graphene nanofilm-based dual-band infrared silicon photodetector. Photon. Res..

[16] Xie J (2023). Zero-bias long-wave infrared nanoantenna-mediated graphene photodetector for polarimetric and spectroscopic sensing. Adv. Opt. Mater..

[17] Zhang L (2023). Ultrahigh-sensitivity and fast-speed solar-blind ultraviolet photodetector based on a broken-gap van der Waals heterodiode. ACS Appl. Mater. Interfaces.

[18] Deng X, Oda S, Kawano Y (2023). Graphene-based midinfrared photodetector with bull’s eye plasmonic antenna. Opt. Eng..

[19] Li K-H (2023). Design strategies toward plasmon-enhanced 2-dimensional material photodetectors. Adv. Dev. AMP Instrum..

[20] Okda HA, Rabia SI, Shalaby HMH (2022). Performance enhancement of an ultrafast graphene photodetector via simultaneous two-mode absorption in a hybrid plasmonic waveguide. Appl. Opt..

[21] Guo J (2022). WSe_2_ /MoS_2_ van der Waals heterostructures decorated with au nanoparticles for broadband plasmonic photodetectors. ACS Appl. Nano Mater..

[22] Yu L (2023). High-bandwidth zero-biased waveguide-integrated p-n homojunction graphene photodetectors on silicon for a wavelength band of 1.55 μm and beyond. ACS Photon..

[23] Wang Y, Fu Y, Zhao J, Liu H, Deng L (2023). Enhanced photoelectric responsivity of graphene/GaAs photodetector using surface plasmon resonance based on ellipse wall grating-nanowire structure. Plasmonics.

[24] Ma Z, Tang P, Xue J, Zhou J (2023). Enhancing photoresponse of GaAs-based photodetector by plasmon grating structures. Plasmonics.

[25] AbdElaziz N, Yousif B, AbdElhalim E, Gaballah WM, Samra AS (2023). Surface plasmonic nano grating for improving GaAs PIN photodetectors performance. Opt. Quantum Electron..

[26] Fan C (2023). Wafer-scale fabrication of graphene-based plasmonic photodetector with polarization-sensitive, broadband, and enhanced response. Adv. Opt. Mater..

[27] Zhang Q (2023). Enhanced gain and detectivity of unipolar barrier solar blind avalanche photodetector via lattice and band engineering. Nat. Commun..

[28] Shen L (2023). Photoresponse improvement of InGaAs nanowire near-infrared photodetectors with self-assembled monolayers. J. Phys. Chem. C.

[29] Park S (2022). Monolithic two-color short-wavelength InGaAs infrared photodetectors using InAsP metamorphic buffers. Appl. Surf. Sci..

[30] Zhou Z (2023). Surface plasmon enhanced InAs-based mid-wavelength infrared photodetector. Appl. Phys. Lett..

[31] Simsek E, Menyuk CR, Subramania GS, Foteinopoulou S (2023). Designing silicon-germanium photodetectors with numerical optimization: The tradeoffs and limits. Active Photonic Platforms (APP) 2023.

[32] Vangelidis I (2022). Unbiased plasmonic-assisted integrated graphene photodetectors. ACS Photonics.

[33] Gosciniak J, Atar FB, Corbett B, Rasras M (2019). Plasmonic Schottky photodetector with metal stripe embedded into semiconductor and with a CMOS-compatible titanium nitride. Sci. Rep..

[34] Liu B (2023). Schottky junction made from a nanoporous Au and TiO_2_ film for plasmonic photodetectors. ACS Appl. Nano Mater..

[35] Li J, Liu J, Guo Z, Chang Z, Guo Y (2022). Engineering plasmonic environments for 2D materials and 2D-based photodetectors. Molecules.

[36] Liu Z (2023). Integrating graphene enables improved and gate-tunable photovoltaic effect in Van der Waals heterojunction. Adv. Opt. Mater..

[37] Abbaszadeh-Azar O, Abedi K (2021). Design of high extinction ratio silicon electro optic modulator based on coupled hybrid plasmonic waveguide using graphene. Superlattices Microstruct..

[38] Viti L, Viti L (2022). Chip-scalable, graphene-based Terahertz thermoelectric photodetectors. 2022 47th International Conference on Infrared, Millimeter and Terahertz Waves (IRMMW-THz).

[39] Xie Z, Wang J, Yeow JTW (2022). Doped Polyaniline/Graphene composites for photothermoelectric detectors. ACS Appl. Nano Mater..

[40] Velusamy DB (2019). MXenes for plasmonic photodetection. Adv. Mater..

[41] Ma P (2019). Plasmonically enhanced graphene photodetector featuring 100 Gbit/s data reception, high responsivity, and compact size. ACS Photon..

[42] Akbari L, Abedi K (2020). A highly sensitive and tunable plasmonic sensor based on a graphene tubular resonator. Opt. Commun..

[43] Moradiani F (2018). Design and analysis of plasmonic switch at mid-IR wavelengths with graphene nano-ribbons. J. Res. Many. Syst..

[44] Abbaszadeh-Azar O, Abedi K (2022). Design of a low power hybrid electro-optic plasmonic modulator based on ITO and graphene. Opt. Mater..

[45] Tiwari SK (2016). Magical allotropes of carbon: Prospects and applications. Crit. Rev. Solid State Mater. Sci..

[46] Bansal S (2022). Bilayer graphene/HgCdTe heterojunction based novel GBn infrared detectors. Micro Nanostruct..

[47] Bansal S (2023). Long-wave bilayer graphene/HgCdTe based GBp type-II superlattice unipolar barrier infrared detector. Results Opt..

[48] Bansal S (2019). Enhanced optoelectronic properties of bilayer graphene/HgCdTe-based single-and dual-junction photodetectors in long infrared regime. IEEE Trans. Nanotechnol..

[49] Kumar R, Jagtap HP, Rajak DK, Bewoor AK, Bhargava C (2020). Traditional and non-traditional optimization techniques to enhance reliability in process industries. AI Techniques for Reliability Prediction for Electronic Components.

[50] Dokeroglu T, Sevinc E, Kucukyilmaz T, Cosar A (2019). A survey on new generation metaheuristic algorithms. Comput. Ind. Eng..

[51] J. Kennedy & R. Eberhart. Particle swarm optimization. In: *Proc. ICNN’95 - International Conference on Neural Networks* vol. 4, 1942–1948 (1995).

[52] Mirjalili SM, Mirjalili S, Lewis A, Abedi K (2014). A tri-objective particle swarm optimizer for designing line defect photonic crystal waveguides. Photon. Nanostruct. Fundam. Appl..

[53] Wang D, Tan D, Liu L (2018). Particle swarm optimization algorithm: An overview. Soft Comput..

[54] Abedi K (2015). Slow light performance enhancement of Bragg slot photonic crystal waveguide with particle swarm optimization algorithm. Opt. Commun..

[55] Mirjalili SM, Abedi K, Mirjalili S (2013). Optical buffer performance enhancement using particle swarm optimization in ring-shape-hole photonic crystal waveguide. Optik.

[56] Schuler S (2021). High-responsivity graphene photodetectors integrated on silicon microring resonators. Nat. Commun..

[57] Li M (2022). Improving performance of hybrid perovskite/graphene-based photodetector via hot carriers injection. J. Alloys Compd..

[58] Yan S, Zuo Y, Xiao S, Oxenløwe LK, Ding Y (2022). Graphene photodetector employing double slot structure with enhanced responsivity and large bandwidth. Opto-Electron. Adv..

[59] Liu X (2022). MoS2-on-GaN plasmonic photodetector using a bowtie striped antenna structure. ACS Appl. Electron. Mater..

[60] Ma Z (2020). Compact graphene plasmonic slot photodetector on silicon-on-insulator with high responsivity. ACS Photon..

[61] Bansal S (2020). A highly efficient bilayer graphene/ZnO/silicon nanowire based heterojunction photodetector with broadband spectral response. Nanotechnology.

[62] Anjum, I. M. *et al.* Use of Evolutionary Optimization Algorithms for the Design and Analysis of Low Bias, Low Phase Noise Photodetectors. *J. Light. Technol.* (2023).

[63] Anjum IM, Simsek E, Mahabadi SEJ, Carruthers TF, Menyuk CR, Anjum IM (2022). Design and analysis of low bias, low phase noise photodetectors for frequency comb applications using particle swarm optimization. 2022 IEEE International Topical Meeting on Microwave Photonics (MWP).

[64] Nag D, Yao W, Van der Tol JJ (2024). Optimization of balanced detector for coherent receiver on generic InP platform by particle swarm optimization. IEEE J. Quantum Electron..

[65] Ferhati H, Djeffal F (2017). A novel high-performance self-powered ultraviolet photodetector: Concept, analytical modeling and analysis. Superlattices Microstruct..

[66] Djeffal F (2021). Highly efficient and low-cost multispectral photodetector based on RF sputtered a-Si/Ti multilayer structure for Si-photonics applications. J. Alloys Compd..

[67] Bencherif H (2023). Plasmon-enhanced Graphene/4H–SiC /graphene metal-semiconductor-metal ultraviolet photodetector: Concept and optimization. Phys. B Condens. Matter.

[68] Simsek E, Anjum IM, Carruthers TF, Menyuk CR (2022). Designing Photodetectors with Machine Learning. In NoW5C-2.

[69] AlAloul, M. & Rasras, M. Supplementary document for Plasmon-enhanced graphene photodetector with CMOS-compatible titanium nitride - 5003830.pdf. doi: 10.6084/m9.figshare.13522196.v2. (2021).

[70] Poli R, Kennedy J, Blackwell T (2007). Particle swarm optimization. Swarm Intell..

[71] Grefenstette J (1986). Optimization of control parameters for genetic algorithms. IEEE Trans. Syst. Man Cybern..

[72] Tang KS, Man KF, Kwong S, He Q (1996). Genetic algorithms and their applications. IEEE Signal Process. Mag..

[73] Al-Madi, N. Binary multi-verse optimization algorithm for global optimization and discrete problems. *Int. J. Mach. Learn. Cybern.*

[74] Bablich A (2022). High-speed nonlinear focus-induced photoresponse in amorphous silicon photodetectors for ultrasensitive 3D imaging applications. Sci. Rep..

[75] Li Z, Yan T, Fang X (2023). Low-dimensional wide-bandgap semiconductors for UV photodetectors. Nat. Rev. Mater..

[76] Xie Z, Wang J, Yeow JTW (2023). Flexible multi-element photothermoelectric detectors based on spray-coated graphene/polyethylenimine composites for nondestructive testing. ACS Appl. Mater. Interfaces.

[77] Zhang Z (2022). Ultraviolet photodetectors based on polymer microwire arrays toward wearable medical devices. ACS Appl. Mater. Interfaces.

[78] Clerc M, Kennedy J (2002). The particle swarm—Explosion, stability, and convergence in a multidimensional complex space. IEEE Trans. Evol. Comput..

[79] Huang GY (2023). Recent progress in waveguide-integrated photodetectors based on 2D materials for infrared detection. J. Phys. Appl. Phys..

[80] Gosciniak J, Khurgin JB (2022). Room temperature plasmonic graphene hot electron bolometric photodetectors: A comparative analysis. J. Appl. Phys..

[81] Chen T (2023). Gate-tunable photovoltaic efficiency in graphene-sandwiched PdSe_2_ photodetectors with restrained carrier recombination. Adv. Opt. Mater..

[82] Lin J (2023). Amorphous-AlZnN/graphene heterostructure for solar-blind ultraviolet photovoltaic detectors. Ceram. Int..

[83] Jia H (2023). Low-noise room-temperature terahertz detector based on the photothermoelectric effect of graphene oxide-Bi films. Opt. Mater..

[84] Li M (2022). Synergistic optimization of photothermoelectric performance of a perovkite/graphene composite. Ceram. Int..

[85] Hong L (2023). Sensitive room-temperature graphene photothermoelectric terahertz detector based on asymmetric antenna coupling structure. Sensors.

[86] Shautsova V (2018). Plasmon induced thermoelectric effect in graphene. Nat. Commun..

[87] Lin Y (2019). Asymmetric hot-carrier thermalization and broadband photoresponse in graphene-2D semiconductor lateral heterojunctions. Sci. Adv..

[88] Mišeikis V (2020). Ultrafast, zero-bias, graphene photodetectors with polymeric gate dielectric on passive photonic waveguides. ACS Nano.

[89] Gosciniak J, Rasras M, Khurgin JB (2020). Ultrafast plasmonic graphene photodetector based on the channel photothermoelectric effect. ACS Photon..

[90] Muench JE (2019). Waveguide-integrated, plasmonic enhanced graphene photodetectors. Nano Lett..

[91] Cheng T, Liu Z, Liu Z, Ding F (2021). The mechanism of graphene vapor–solid growth on insulating substrates. ACS Nano.

[92] Liu J-M, Lin I-T (2018). Graphene Photonics.

[93] Li C (2024). Exploiting the correlation between 1/f noise-dark current in PIN InGaAs photodetectors. IEEE J. Quantum Electron..

[94] Wang J, Wu S, Mi C, Qiu Y, Bai X (2024). A low-noise, high-gain, and large-dynamic-range photodetector based on a JFET and a charge amplifier. Front. Inf. Technol. Electron. Eng..

[95] Sun T (2024). Gain and excess noise properties of 3-gain-stage InGaAs/InAlAs avalanche photodetector. Phys. B Condens. Matter.

[96] Crisci T, Moretti L, Casalino M (2021). Theoretical investigation of responsivity/NEP trade-off in NIR graphene/Semiconductor Schottky photodetectors operating at room temperature. Appl. Sci..

